# Protists are key players in the utilization of protein nitrogen in the arbuscular mycorrhizal hyphosphere

**DOI:** 10.1111/nph.70153

**Published:** 2025-04-22

**Authors:** Anukool Vaishnav, Martin Rozmoš, Michala Kotianová, Hana Hršelová, Petra Bukovská, Jan Jansa

**Affiliations:** ^1^ Laboratory of Fungal Biology, Institute of Microbiology Czech Academy of Sciences Vídeňská 1083 14200 Prague 4 Czech Republic

**Keywords:** arbuscular mycorrhizal fungus, hyphosphere, multitrophic interactions, organic nitrogen, quantitative real‐time PCR, stable isotopes, temporal dynamics

## Abstract

- While largely depending on other microorganisms for nitrogen (N) mineralization, arbuscular mycorrhizal fungi (AMF) can transfer N from organic sources to their host plants. Here, we compared N acquisition by the AMF hyphae from chitin and protein sources and assessed the effects of microbial interactions in the hyphosphere.
- We employed *in vitro* compartmented microcosms, each containing three distinct hyphosphere compartments amended with different N sources (protein, chitin, or ammonium chloride), one of which was enriched with ^15^N isotope. All hyphosphere compartments were supplied with *Paenibacillus* bacteria, with or without the protist *Polysphondylium pallidum*. We measured the effect of these model microbiomes on the efficiency of ^15^N transfer to roots via the AMF hyphae.
- We found that the hyphae efficiently took up N from ammonium chloride, competing strongly with bacteria and protists. Mobilization of ^15^N from chitin and protein was facilitated by bacteria and protists, respectively. Notably, AMF priming significantly affected the abundance of bacteria and protists in hyphosphere compartments and promoted mineralization of protein N by protists. Subsequently, this N was transferred into roots.
- Our results provide the first unequivocal evidence that roots can acquire N from proteins present in the AMF hyphosphere and that protists may play a crucial role in protein N mineralization.

## Introduction

Nitrogen (N) is usually the most limiting mineral nutrient among those that plants obtain from the soil, and its availability often determines growth and yields of crops (Tegeder & Masclaux‐Daubresse, 2018). For over a century, agronomists have held the belief that crop plants primarily absorb N in the form of nitrate (NO_3_^−^) and/or ammonium (NH_4_^+^), while the uptake of organic N was only considered in natural environments, such as arctic or forested ecosystems, where inorganic N generally is scarce (Wang *et al*., 2018). This belief as well as the availability of industrially produced mineral N fertilizers has led to a reliance on inorganic N fertilizers in agriculture, with most studies targeting inorganic N dynamics rather than organic N. Although the widespread use of inorganic N fertilizers has resulted in significant yield improvements, it has also caused soil degradation and environmental pollution at unprecedented scales (Lassaletta *et al*., 2014; Hagh‐Doust *et al*., 2023). A substantial portion (up to 50%) of the inorganic N applied in agriculture is not utilized by crops and, after undergoing various transformations, can become a serious pollutant in water and air (Coskun *et al*., 2017). This pollution disrupts regional biogeochemical N cycles as well as the cycling of carbon (C) and other elements (Grandy *et al*., 2022).

In recent decades, ecological and agricultural studies have suggested that the traditional view of plant N nutrition is overly simplistic. The importance of organic N in agricultural crop production has often been overlooked (Näsholm *et al*., 2009; Farzadfar *et al*., 2021). Organic N forms constitute 80% to 90% of the total N pool in the soil, except that immediately after the application of inorganic N fertilizers, the inorganic N fraction temporarily increases (Liu *et al*., 2018). Soil organic N dynamics have been studied with several model and crop plants, including *Plantago*, wheat, barley, maize, clover, and sugarcane (Hodge *et al*., 2001; Jämtgård *et al*., 2008; Czaban *et al*., 2016; Enggrob *et al*., 2019; Farzadfar *et al*., 2021). This has enhanced our understanding that plants can absorb a variety of N forms, including both inorganic and organic (as oligomers or monomers). However, agricultural systems typically receive external N inputs that create conditions for N cycling different from those in natural systems. This discrepancy has generated a crucial research gap concerning the availability and uptake mechanisms of organic N for plant use in agricultural systems (Thirkell *et al*., 2016). To address this gap, we need a deeper understanding as to the role of soil organic N in plant N budgets. Efficient utilization of soil organic N can reduce agriculture's overreliance on inorganic fertilizers and promote regenerative agriculture practices. These practices could in turn improve the efficiency of fertilizer N use and mitigate agriculture's negative impact on the environment (Farzadfar *et al*., 2021).

In agricultural soils, the primary sources of organic N include manure, crop residues, roots, root exudates, and microbial necromass. Proteins and amino sugars contained in these sources serve as the main forms of N for microbes, plants, and animals (Holz *et al*., 2024). Microbes depolymerize the biopolymers into oligomers and monomers, which constitute those forms of organic N most accessible for plants (Paungfoo‐Lonhienne *et al*., 2008; Näsholm *et al*., 2009). These forms undergo further processes, as well, such as deamination, which releases free ammonium ions into the soil solution (a process sometimes referred to as N mineralization or ammonification). Current understanding suggests that in ecosystems with slow microbial N mineralization, plants rely on ecto‐ or ericoid mycorrhizal fungal symbioses to break down organic compounds (Adamczyk, 2021). Conversely, in ecosystems with high microbial activity and N mineralization rates, plants often depend on arbuscular mycorrhizal fungi (AMF) (Adamczyk, 2021). While AMF cannot independently break down organic matter, previous research has shown that they rely on the activity of other soil microorganisms to access organic N in the vicinity of their hyphae (Tisserant *et al*., 2013; Jansa *et al*., 2018; Hestrin *et al*., 2019). Studies have indicated that both primary decomposers and their grazers, such as soil protists, likely play important roles in making soil organic N available for uptake by AMF and facilitating its transport to plants (Koller *et al*., 2013; Bukovská *et al*., 2018; Henkes *et al*., 2018; Rozmoš *et al*., 2022). In conditions of low N availability, however, competition arises for N among AMF, plants, and other soil microorganisms (Kuzyakov & Xu, 2013), potentially leading to negative effects of AMF on plant N acquisition (Herdler *et al*., 2008; Püschel *et al*., 2016).

Studying multitrophic interactions (including AMF, bacteria, and protists) may enhance our understanding of organic N's mineralization and its further assimilation into plants. Nevertheless, such studies present challenges due to a lack of experimental systems that can conclusively differentiate between functioning of fungal symbionts and other associated organisms (Hoysted *et al*., 2023). This distinction is significant, because increasing evidence indicates that the AMF hyphae associate with various nutrient‐mineralizing microbes and specific protists in the hyphosphere, where they may compete for nutrients originating from soil organic sources (Kuzyakov & Xu, 2013; Jansa *et al*., 2018; Duan *et al*., 2024; Zhang *et al*., 2024). Recent experiments from our group have improved our understanding of how plants acquire N from organic sources in the soil, particularly through AMF, with the help of hyphosphere‐associated bacteria and protists. In Bukovská *et al*. (2018), we demonstrated that the extraradical AMF hyphae in the presence of complex soil microbial communities can obtain a large share of N from chitin and clover biomass and transfer it to plants. We found that the AMF hyphae showed greater colonization of organic patches than those receiving only mineral nutrient sources. However, the experimental design did not allow to gain further insights into the interplay between specific hyphosphere microbes and the AMF hyphae with respect to exploration of organic N sources. Later, in Rozmoš *et al*. (2022), we showed that the presence of chitinolytic bacteria, alone or with protists, in the AMF hyphosphere enhanced N uptake by the plant from chitin. We also observed that some strains of bacteria positively affected the development of the AMF hyphae in the chitin compartment. Remaining to be tested, however, was the reverse interaction, which is to say how AMF influence the abundance of other microorganisms in organic patches. The aforementioned findings raised several questions: How do the AMF hyphae influence the growth of bacteria and protists in the hyphosphere? Do such effects facilitate utilization of organic N by the hyphae? Do the hyphae have access to different N compounds from the hyphosphere, considering that soil organic N is chemically heterogeneous? If so, to what extent do different organisms influence access to those various compounds?

In this study, we aimed to address these research questions by utilizing a monoxenic symbiotic culture of *Rhizophagus irregularis* (Błaszk., Wubet, Renker & Buscot; C. Walker & A. Schüßler), associated with Ri T‐DNA‐transformed chicory (*Cichorium intybus* L.) roots. We employed stable ^15^N isotope labeling to track the transfer of N from protein and chitin, as compared to inorganic N (NH_4_Cl), from a root‐free patch to the roots via the mycorrhizal (hyphal) pathway while also considering the presence of bacteria (*Paenibacillus chitinolyticus* Kuroshima *et al*., 1996; Lee *et al*., 2004) and a protist (*Polysphondylium pallidum* Olive) in the hyphosphere. We sought to test two specific hypotheses: (1) Introducing bacteria with both chitinolytic and proteolytic capabilities, along with protists, into the hyphosphere supplied with organic N would enhance the rates of N release from chitin and protein. This, in turn, would lead to improved N uptake from the organic pool by the AMF hyphae and subsequent transfer of such N to the roots. (2) The presence of AMF would increase the abundance of bacteria and protists in the hyphosphere. To test these hypotheses, we designed a novel microcosm system with a root compartment and three root‐free (hyphosphere) compartments amended with different N sources. Only the AMF hyphae were permitted to make contact with the hyphosphere compartments. We also manipulated the presence of protists in these compartments to evaluate their role in N mineralization and/or acquisition by AMF from various N sources.

## Materials and Methods

### Biological materials

We utilized a monoxenic culture of *R. irregularis* LPA9 (= BEG 236), which has been maintained *in vitro* in association with Ri T‐DNA‐transformed chicory roots for several years at the Laboratory of Fungal Biology, Institute of Microbiology of the Czech Academy of Sciences (Prague). To establish a non‐mycorrhizal (NM) control, we used the same root culture but without AMF (referred to as NM roots). The bacterium *P. chitinolyticus* CCM 4527 employed in a previous study (Rozmoš *et al*., 2022) was also used in the experiment described here. The chitinase and protease activities of this bacterium were confirmed on chitin and casein‐amended media, respectively (Supporting Information Fig. S1). Additionally, we included the protist *P. pallidum* (Amoebozoa) in this study. It had originally been isolated from spruce bark compost (Bukovská *et al*., 2016) and maintained with *Escherichia coli* coculture on lysogeny broth agar (1.5%) for several years.

### ^15^N‐labeled N sources

In this study, we utilized three different ^15^N‐labeled N sources: (1) ammonium chloride (NH_4_Cl, AT% ^15^N = 99%) purchased from Cambridge Isotope Laboratories Inc. (Tewksbury, MA, USA); (2) chitin; and (3) leaf protein. The ^15^N‐labeled chitin was prepared from the cell walls of *Zygorhynchus* sp. as previously described by Bukovská *et al*. (2018). The ^15^N‐labeled leaf protein was extracted from clover leaves grown with ^15^N‐labeled potassium nitrate (AT% ^15^N = 99%, Cambridge Isotope Laboratories) according to a protocol previously described by Niu *et al*. (2018). Briefly, after harvesting, 2 g of fresh clover leaf biomass was crushed in liquid N. Proteins were then extracted from the powdered biomass using 10 ml of extraction buffer, which contained 1% SDS, 1 M Tris–HCl, 2 mM EDTA‐Na_2_, and 20 mM DTT (pH = 8.0), along with a few glass beads. The mixture was vortexed and centrifuged at 3700 ***g*** for 15 min at 4°C. The supernatant was collected in a fresh Falcon tube and precipitated using a 20% TCA/acetone solution (1/1, v/v) at −20°C for *c*. 20–30 min. The mixture was centrifuged again at 3700 ***g*** for 15 min at 4°C. The supernatant was discarded, and the pellet was resuspended in chilled 80% acetone and left at −20°C for 20 min. This last step was repeated two more times. Finally, the pellet was resuspended in chilled absolute acetone and left overnight at −20°C. Afterward, it was centrifuged at 3700 ***g*** for 15 min at 4°C, and the supernatant was removed. The pellet was then allowed to dry at room temperature.

### Compartmentalized microcosm setup and conducting the experiment

**Fig. 1:**
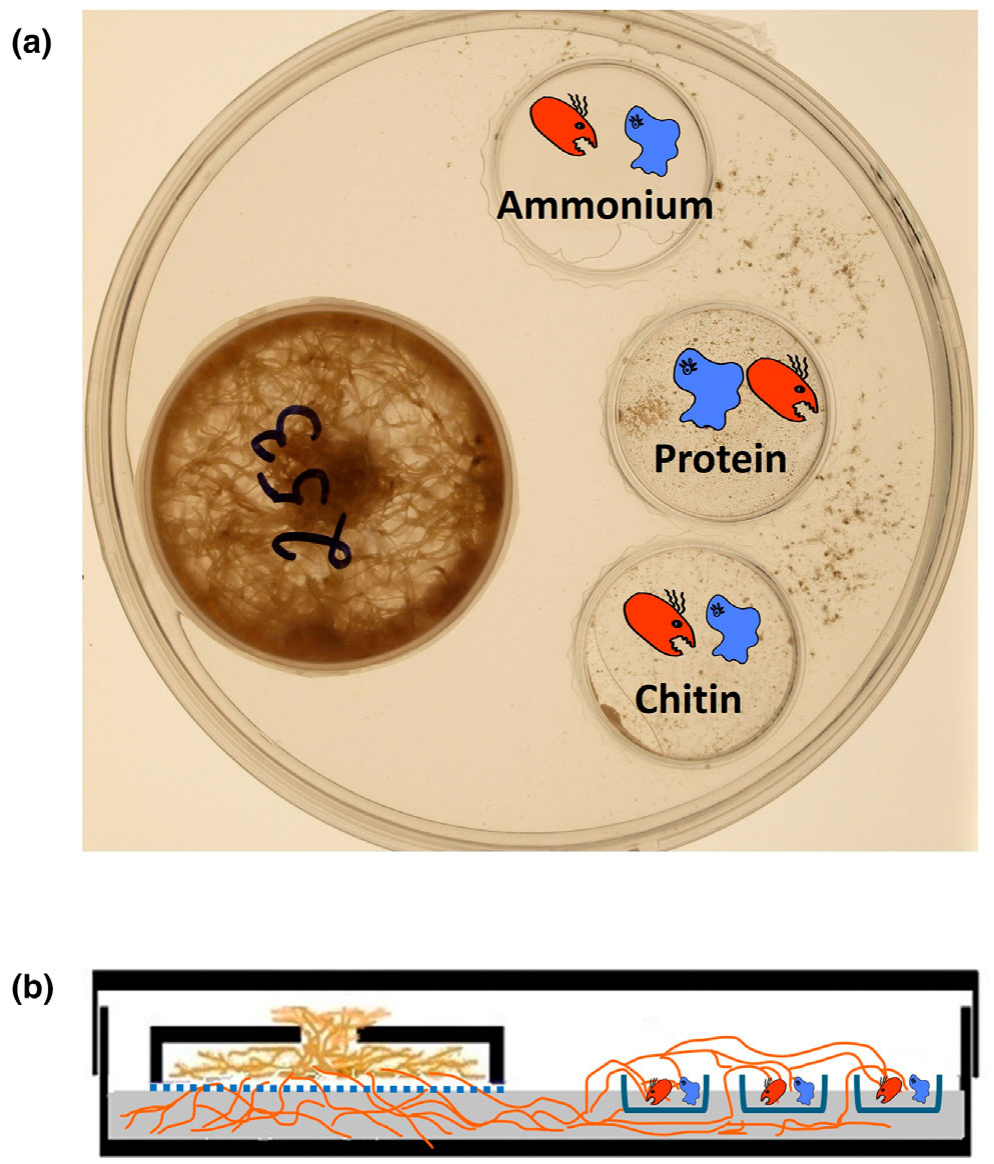
Design of a compartmented microcosm showing root compartment and three root‐free (hyphosphere) compartments per microcosm. (a) Experimental microcosm consisting of a root compartment and three hyphosphere compartments amended with various nitrogen (N) sources. The microcosms were each created of a large Petri dish 15 cm in diameter and with a height of 2 cm. Inside of this dish was placed a small root compartment (6 cm in diameter, fitted with a 40‐μm mesh at the bottom to contain the roots) and three hyphosphere compartments (3.5 cm in diameter). The root compartment contained the Ri T‐DNA‐transformed roots of *Cichorium intybus* L., which could either be nonmycorrhizal or mycorrhizal with *Rhizophagus irregularis* (Błaszk., Wubet, Renker & Buscot) C. Walker & A. Schüßler, isolate LPA9. Each of the three hyphosphere compartments was filled with a modified Strullu and Romand (MSR) medium that was free of N and sucrose, but supplemented with chitin, ammonium chloride, or protein as N sources individually in each compartment. One isotopically (^15^N) labeled N source at a time was used in each setup. All three hyphosphere compartments were inoculated with *Paenibacillus chitinolyticus* (Kuroshima *et al*., 1996; Lee *et al*., 2004) isolate CCM4527 (shown in red), and a protist *Polysphondylium pallidum* Olive (shown in blue) was either present or absent according to the treatment. (b) Front view of the complete setup, illustrating the arbuscular mycorrhizal fungus hyphae (orange threads) growing out of the roots through the 40‐μm mesh (blue dotted line), colonizing the MSR medium that filled the volume of the large Petri dish, and eventually reaching the hyphosphere compartments, the rims that were protruding above the MSR medium, forming a diffusion barrier for water and solutes.

This experiment was conducted to evaluate the capacity of *R. irregularis* LPA9 to explore, utilize, and transport ^15^N back to the roots from various isotopically labeled N sources supplied within the hyphosphere in the presence of bacteria and with or without the protist. The experiment was carried out in an *in vitro* microcosm setup similar to one used previously (Rozmoš *et al*., 2022). It consisted of a large sterile polystyrene Petri dish (diameter 15 cm, height 2 cm) containing four smaller compartments (Fig. 1). One of the smaller compartments, referred to as the root compartment, was made from the lid of a small (6 cm diameter) polystyrene Petri dish, which had a hole (12 mm in diameter) drilled into the top for root transfer. The bottom opening was sealed with a 40‐μm nylon mesh (Silk & Progress, Brněnec, Czech Republic). To secure the mesh to the lid rim, that rim was dipped in toluene, then pressed against the mesh, firmly gluing the lid rim to the mesh. After preparing the root compartments, they were sterilized using gamma rays (> 25 kGy; Bioster, Veverská Bítýška, Czech Republic). The remaining three smaller (root‐free) compartments containing different N forms were created from the bottoms of three small (35 mm diameter) Petri dishes and positioned at least 5 mm distant from the root compartment. The large Petri dish was filled with 100 ml of the standard modified Strullu and Romand (MSR) medium (pH 5.5), which contained 93 μg of phosphorus (P) and 379 μmol of N, along with 1% (w/v) sucrose and solidified using 0.3% (w/v) gelling agent (Phytagel; Merck, Darmstadt, Germany) as described previously (Cranenbrouck *et al*., 2005). The concentrations of P and N in the Phytagel powder were 560 μg g^−1^ and 46 μmol g^−1^, respectively (Rozmoš *et al*., 2022). Sterile root‐free compartments were placed in an empty 150‐mm Petri plate and held down with metal plugs before the MSR medium was poured around them. Once the medium had solidified, the metal plugs were removed, and the root compartment was placed onto the surface of the solid medium (Fig. 1).

Mycorrhizal and NM chicory roots grown previously on the solid MSR medium were chopped into segments *c*. 5–10 mm in length, including the medium (and the AMF hyphae/spores, if applicable). These were then added as inoculum in the root compartment through the drilled hole. The setup was incubated at 24°C in darkness for 56 d. By the end of this incubation period, the roots had filled the root compartment, and the AMF hyphae had colonized the entire volume of the MSR medium. On the 57^th^ day, three different types of N sources were added to the hyphosphere compartments: 15 mg of chitin, either ^14^N or ^15^N (C = 44% by weight and N = 3.8 μmol mg^−1^, with AT% ^15^N = 0.363% for the ^14^N; and C = 45% by weight and N = 3.7 μmol mg^−1^, with AT% ^15^N = 99% for the ^15^N variant), 15 mg protein, either ^14^N or ^15^N (C = 47.7% by weight and N  = 10.5 μmol mg^−1^, with AT% ^15^N = 0.368% for the ^14^N; and C = 47.6% by weight and N = 10.5 μmol mg^−1^, with AT% = 94.8% for the ^15^N variant), or NH_4_Cl (supplying always 57.2 μmol N either as ^14^N or ^15^N, with AT% values being 0.365% and 99% for the ^14^N and ^15^N variants, respectively). One of the compartments per each microcosm was supplied with a ^15^N‐labeled N source, whereas the others contained nonlabeled N sources. The different N sources were provided in 5 ml of the N‐free MSR medium without sucrose and containing 0.3% Phytagel, in a procedure similar to that described previously by Rozmoš *et al*. (2022) and detailed in Fig. S2. After an additional 19 d of incubation (on the 76^th^ day), 60 μl of medium containing actively growing *P. chitinolyticus* CCM4527 (48–h‐old culture) was added to each hyphosphere compartment. Following another 17 d (on the 93^rd^ day), a suspension of *P. pallidum* spores grown on the same bacterial culture (*P. chitinolyticus* CCM4527) as described previously was added to all three hyphosphere compartments in half of the microcosms. The microcosms were then incubated for an additional 15 d (see Fig. S2 for a detailed timeline).

The experiment was laid out as a fully factorial, with seven replicate microcosms for each treatment combination, each of which included three factors: (1) presence of AMF (two levels: present or absent); (2) type of ^15^N amendment in the hyphosphere compartment (three levels: chitin, NH_4_Cl, or protein); and (3) presence of protist (two levels: present or absent). Bacteria were always added into all three hyphosphere compartments. In total, 84 microcosms were set up for this experiment. Further details can be found in Fig. S2.

### Harvesting and analyses

At 108 d after inoculation, the roots were collected and dried at 65°C. The dried roots were weighed and pulverized in a ball mill (MM 200; Retsch, Haan, Germany). Next, the N concentrations and isotopic compositions of N were measured using a Flash 2000 elemental analyzer coupled with a Delta V Advantage isotope‐ratio mass spectrometer (Thermo Fischer Scientific, Bremen, Germany). For this analysis, 2 mg of aliquots was wrapped in tin capsules. The AMF hyphae were extracted from the root‐free compartment (i.e. the large dish volume) by dissolving Phytagel in potassium citrate buffer (10 mM, pH 6.0), as described previously by Rozmoš *et al*. (2022). The hyphae were then collected on an Omnipore membrane filter (5‐μm pore size, 47 mm diameter; Merck Millipore, Burlington, MA, USA). The weight of the hyphae was recorded after drying in a vacuum centrifuge for 2 d, and N concentration and N isotopic composition were analyzed in the same manner as for the roots.

The contents of the hyphosphere compartments were frozen and lyophilized before DNA extraction, as previously described (Rozmoš *et al*., 2022). The samples were homogenized in ceramic mortars, spiked with an internal DNA standard (Thonar *et al*., 2012), and the DNA was extracted using the glass‐milk method (Gryndler *et al*., 2013). The DNA extracts were then used as templates in quantitative real‐time PCR (qPCR), employing primers and hydrolysis (TaqMan) probes that specifically targeted the internal DNA standard, the mitochondrial large ribosomal subunit rRNA gene of *R. irregularis*, the 18S rRNA gene of *P. pallidum*, and the eubacterial 16S rRNA gene using a Lightcycler II (Roche, Rotkreuz, Switzerland). Primer and probe sequences, cycling conditions, and quantification methods followed those of Dudáš *et al*. (2022) and Rozmoš *et al*. (2022).

### Data calculation and statistical analyses

The N contents of the roots and the AMF hyphae from each microcosm were determined based on the respective N concentrations and dry biomass of the samples. To calculate the excess ^15^N values (representing the amounts of N originating from the isotopically enriched inputs), molar N concentrations in the samples and their ^15^N abundances expressed as atom% were considered. The ^15^N abundance of additional samples without any added ^15^N‐labeled compounds served as an isotopic baseline. The percentage of ^15^N transfer from the labeled root‐free compartment to the AMF hyphae and/or roots was calculated using a two‐source mixing model based on mass balance equations, as detailed by Phillips & Gregg (2001). Knowing the isotopic enrichment levels of the labeled compounds (as discussed earlier) and their corresponding inputs per experimental system, we determined the fraction of ^15^N supplied per microcosm recovered in either the roots or the AMF hyphae outside of the hyphosphere compartments. Data analyses were performed using one‐way, two‐way, and three‐way analyses of variance (ANOVA), followed by Tukey's *post hoc* test to separate treatment means if ANOVA results were significant (*P* < 0.05). These analyses were conducted using R 4.4.1 (R Development Core Team, 2024). The microbial abundance data were log‐transformed before statistical analyses. To evaluate the effects of different factors on microbial abundance in the different root‐free compartments (inasmuch as three measurements were obtained per each microcosm, one for each of the three root‐free compartments), we used linear mixed‐effects models with microcosm identity as a random effect and as implemented by the ‘lme’ function in the ‘nlme’ package (Pinheiro *et al*., 2023). ANOVA results are presented in Tables 1 and S1. Absolute values presented later are treatment mean ± SE of the mean.

**Table 1:** Result of three‐way analyses of variance assessing the fraction of ^15^N transported out of the ^15^N‐labeled hyphosphere compartments and detected in the roots and the AMF hyphae outside of the labeled compartments.

Factor	Degrees of freedom	Mean square	*F‐*ratio	*P*‐value
AMF	1	5894	1219	< 0.001
Protist	1	42	8.73	0.004
N source	2	2307	477	< 0.001
AMF × Protist	1	23	4.81	0.032
AMF × N source	2	1077	223	< 0.001
Protist × N source	2	13	2.59	0.082
AMF × Protist × N source	2	19	4.0	0.022
Residuals	71	5		

AMF, arbuscular mycorrhizal fungi; N, nitrogen.

## Results

### Proliferation of the AMF hyphae in the hyphosphere compartments

Several weeks after setting up the experiment, AMF began to proliferate outside of the root compartment, forming a fine hyphal network (Fig. S3a). Once the different N sources had been added to their respective hyphosphere compartments, the AMF hyphae started to enter those compartments within a few days. Microscopic observation revealed rapid colonization characterized by the long finely branched AMF hyphae and some spores on NH_4_Cl. By contrast, the growth of the hyphae on chitin and protein was slower and exhibited different morphology, featuring long and thin hyphae and dispersed branching (Fig. S3b–d). Following the addition of the protist, it showed early maturation with fruiting bodies and whorled sorocarps forming as early as 3 d after inoculation in ammonium and protein patches, while fruiting bodies started to appear only as of the 4^th^ day from inoculation on chitin media (Fig. S4).

### Transfer of ^15^N from the hyphosphere compartments to the roots

**Fig. 2:**
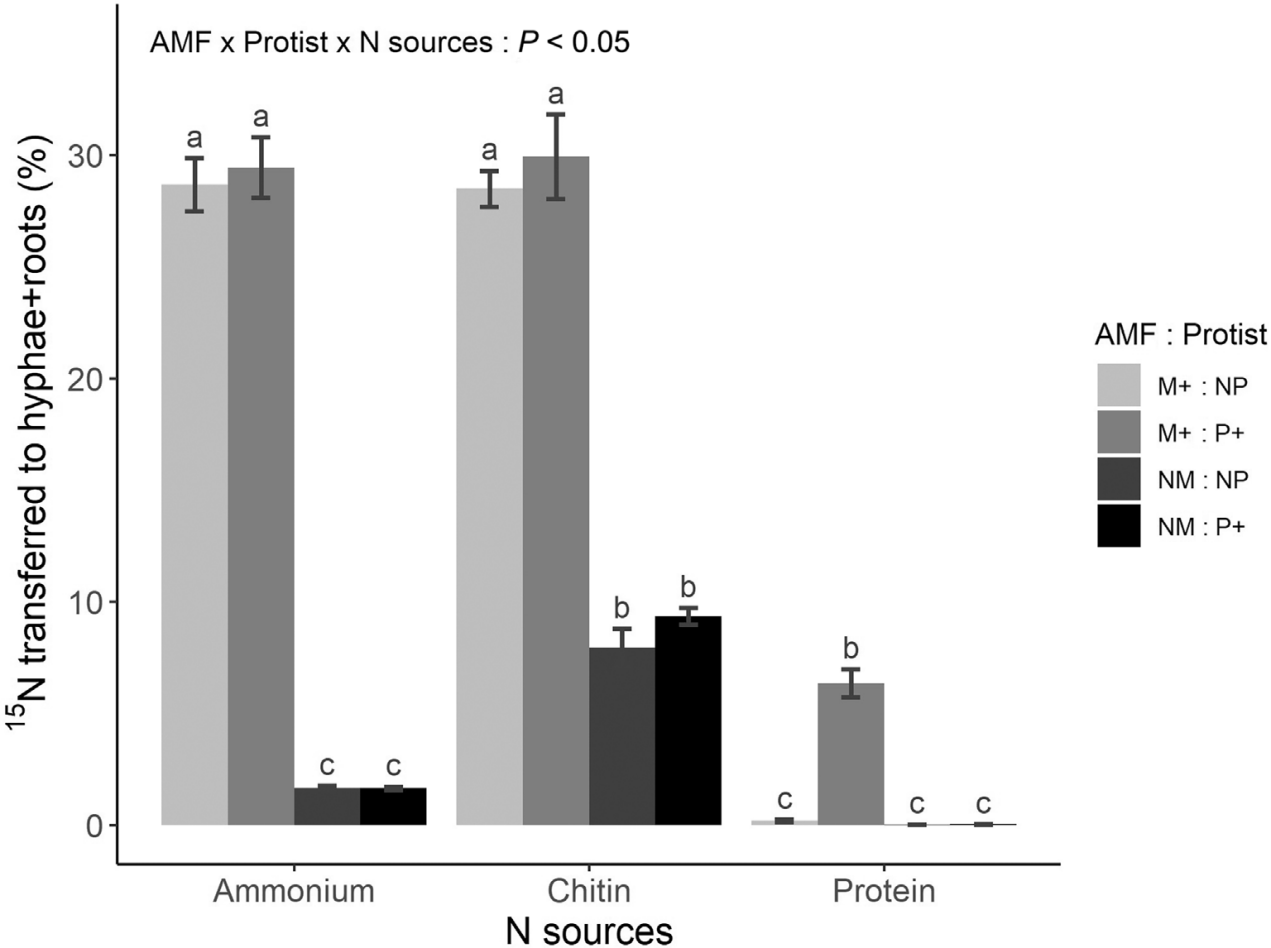
Fraction of ^15^ nitrogen (N) amount supplied into the hyphosphere compartment in different chemical forms and detected in the *Cichorium intybus* roots and mycorrhizal hyphae outside of the hyphosphere compartments. The different forms of N were ammonium (NH_4_Cl), chitin, or protein. Effects from the presence of arbuscular mycorrhizal fungus *Rhizophagus irregularis* LPA9 (AMF) and protist *Polysphondylium pallidum* are illustrated (M+, mycorrhizal treatment; NM, nonmycorrhizal treatment; P+, protists present; NP, protists absent). The results are presented as mean values ± SE (*n* = 7) of the total ^15^N‐transfer values (i.e. the excess ^15^N contained in the roots + excess ^15^N in the AMF hyphae collected from the main Petri dish divided by total excess ^15^N supplied per isotopically labeled hyphosphere compartment, ×100). Different letters indicate significant differences between treatment means as per Tukey's *post hoc* test (*P* < 0.05), following a significant three‐way analysis of variance (significance of the three‐way interaction is provided).

The transfer of ^15^N from the hyphosphere compartments was significantly influenced by all three experimental factors: the presence of AMF (*F*_1,71_ = 1219, *P* < 0.001), the presence of protists (*F*_1,71_ = 8.73, *P* < 0.01), and the chemical form in which ^15^N was supplied (*F*_2,71_ = 477, *P* < 0.001) (Fig. 2). The largest transfer, *c*. 30% of the added ^15^N, was observed in the mycorrhizal microcosms from the ammonium and chitin sources. In NM systems, by contrast, only *c*. 1.5% and 9% were transferred from ammonium and chitin, respectively. The transfer of ^15^N from ammonium and chitin was not significantly affected by the presence of protists, although it was nevertheless somewhat higher when protists were present. The uptake of ^15^N from proteins increased significantly in the presence of protists in mycorrhizal roots, whereas no such effect was observed in the absence of AMF, and this was reflected in a significant protist × AMF interaction term (Fig. 2; Table 1). When protists were absent, roots and their associated AMF hyphae in the mycorrhizal microcosms took up less than 1% of the added ^15^N in the form of protein. When protists were present, however, the uptake increased to *c*. 6% (summing up the ^15^N in both the roots and their associated hyphae). The uptake of protein N in NM roots was below 0.05% of the ^15^N added regardless of the presence or absence of protists.

### Allocation of ^15^N in roots and hyphae

**Fig. 3:**
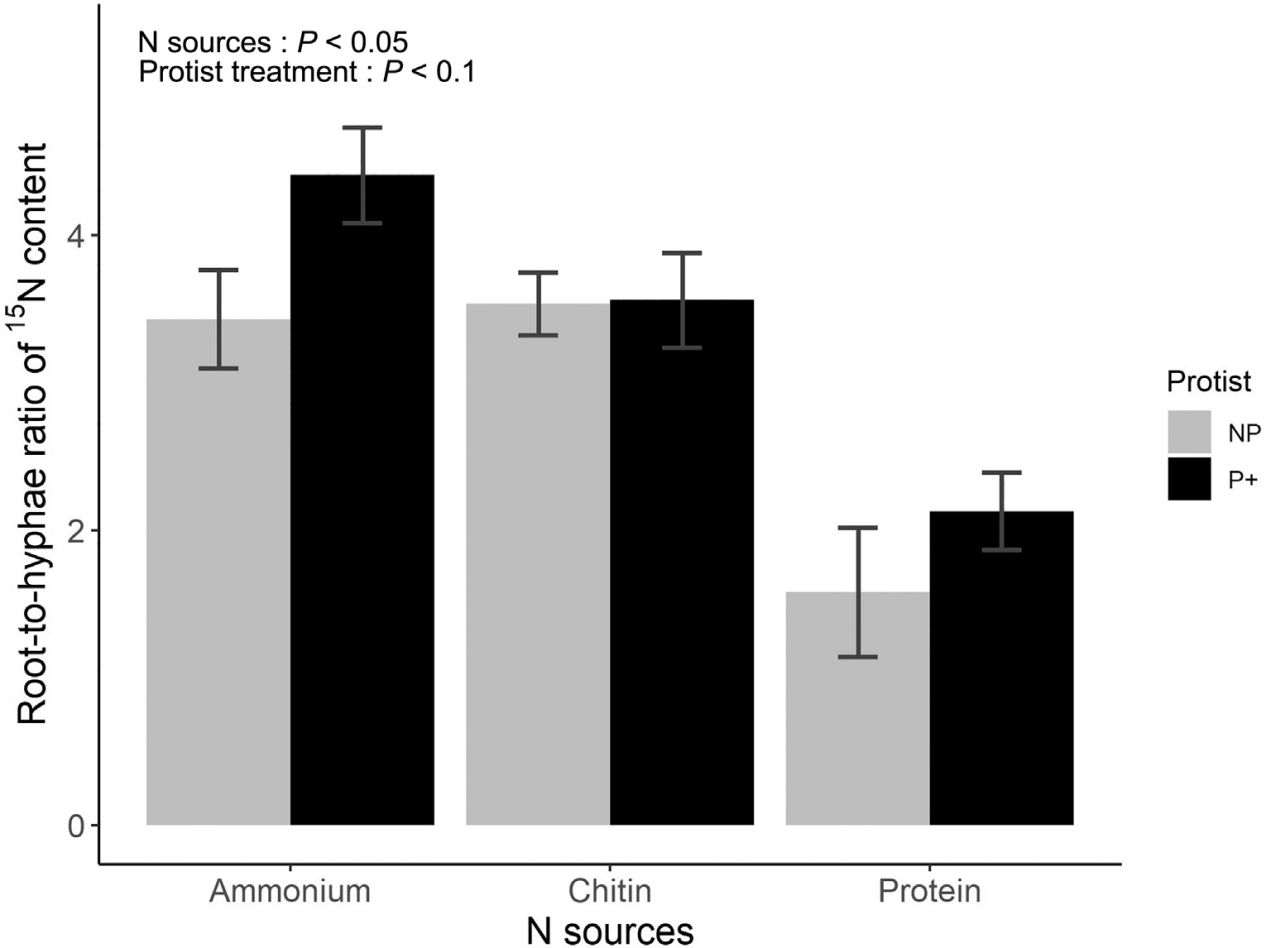
Impact of protist *Polysphondylium pallidum* presence on ^15^ nitrogen (N) partitioning between *Cichorium intybus* roots and mycorrhizal hyphae of *Rhizophagus irregularis* LPA9 depending upon the chemical form of the labeled N sources. The different forms of N were ammonium (NH_4_Cl), chitin, and protein. This analysis was conducted using the data from mycorrhizal microcosms only. The results are presented as mean values ± SE (*n* = 7) for the ratio of excess ^15^N contained in roots to the ^15^N excess contained in the mycorrhizal hyphae collected from the main Petri dish. P+, protists present; NP, protists absent. Significance of the individual factors is provided; the interaction between the factors was not statistically significant.

To investigate the distribution of ^15^N sourced from different N forms between roots and the AMF hyphae, we calculated the root‐to‐hyphae ratio of ^15^N for the mycorrhizal treatment only. The distribution of ^15^N between roots and hyphae was not only significantly influenced by the type of N source (*F*_2,35_ = 23.1, *P* < 0.001) but also marginally by the presence of protists (*F*_1,35_ = 3.11, *P* < 0.1), but not by their interaction (Fig. 3; Table S1). The highest root‐to‐hyphae ratio for ^15^N distribution was observed for the NH_4_Cl treatment (4.4 ± 0.3), whereas the lowest ratio was recorded for proteins (1.6 ± 0.4).

### Microbial abundance in the hyphosphere compartments

**Fig. 4:**
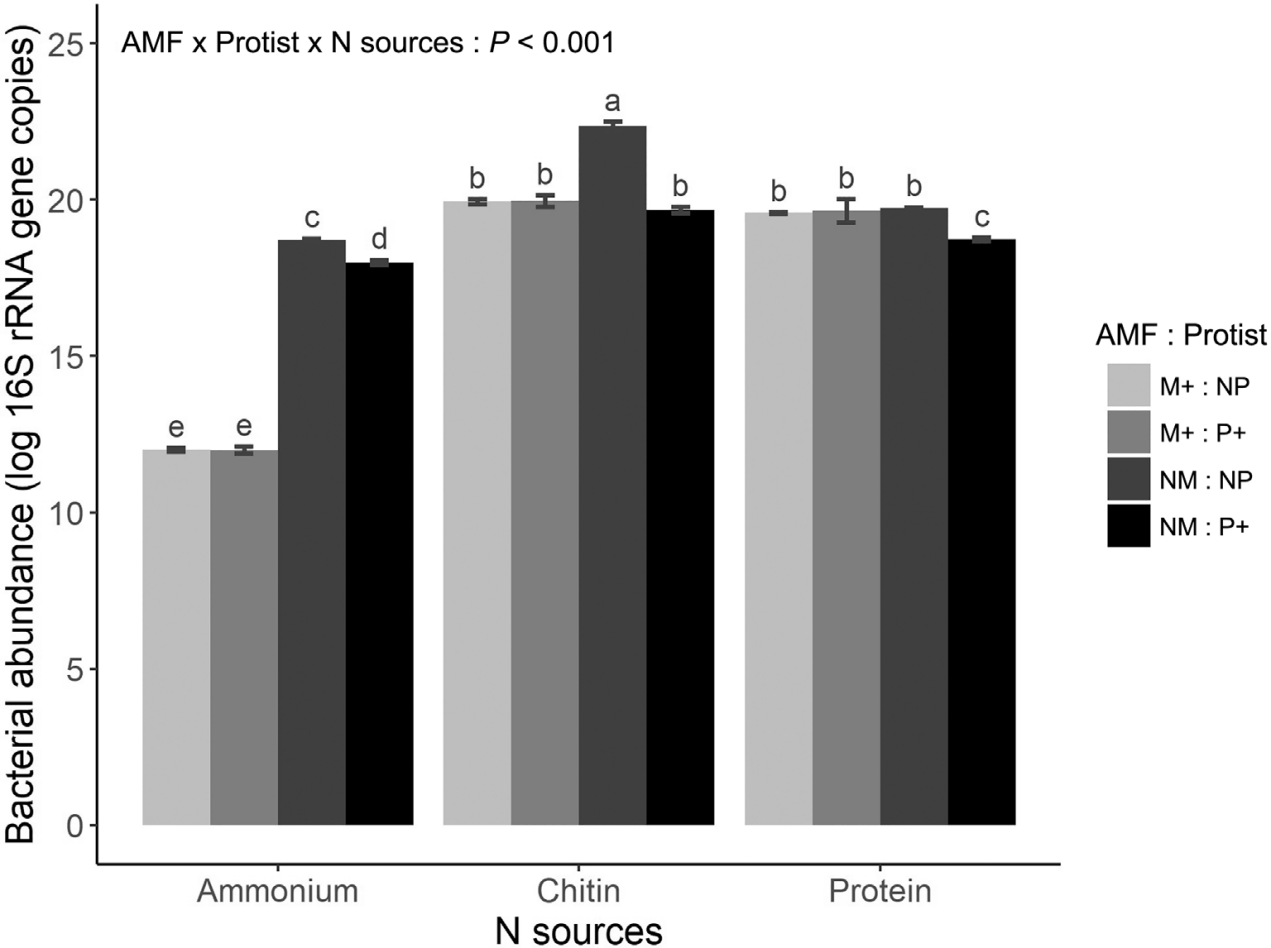
Abundance of bacterium *Paenibacillus chitinolyticus* CCM4527 in the three hyphosphere compartments at final harvest as assessed by quantitative real‐time PCR (qPCR). The qPCR primers targeted the 16S rRNA gene. Effects from the presence of arbuscular mycorrhizal fungus *Rhizophagus irregularis* LPA9 (AMF) and protist *Polysphondylium pallidum* are illustrated (M+, mycorrhizal treatment; NM, non‐mycorrhizal treatment; P+, protists present; NP, protists absent) depending upon the chemical form of nitrogen (N) source added to each hyphosphere compartment. The different forms of N were ammonium (NH_4_Cl), chitin, and protein. Data were log‐transformed before analysis. A linear mixed‐effects analysis of variance (ANOVA) model with microcosm identity as random effect was employed to acknowledge potential codependency of analyses of three root‐free compartments from the same microcosm. The results are presented as mean values ± SE (*n* = 21). Different letters indicate significant differences between treatment means as per Tukey's *post hoc* test (*P* < 0.05) following a significant three‐way ANOVA (significance of the three‐way interaction is provided).

The abundance of bacteria and protists was significantly influenced by the presence of AMF and the types of N sources used. Bacterial abundance was considerably affected by all three factors and their interaction (*F*_2,158_ = 18.0, *P* < 0.001 for the three‐way interaction; Table S1). The greatest bacterial abundance was observed with chitin (5.6 × 10^9^ ± 3.0 × 10^8^ 16S gene copies per root‐free compartment), followed by protein (3.7 × 10^8^ ± 8.2 × 10^6^ 16S gene copies per root‐free compartment), and NH_4_Cl (1.4 × 10^8^ ± 5.4 × 10^6^ 16S gene copies per root‐free compartment) when AMF and protists were absent in the root‐free compartments (Fig. 4). In the presence of protists, bacterial abundance generally decreased. Nevertheless, it remained unaffected by protists when AMF were also present alongside protists in all three compartments (Fig. 4).

**Fig. 5:**
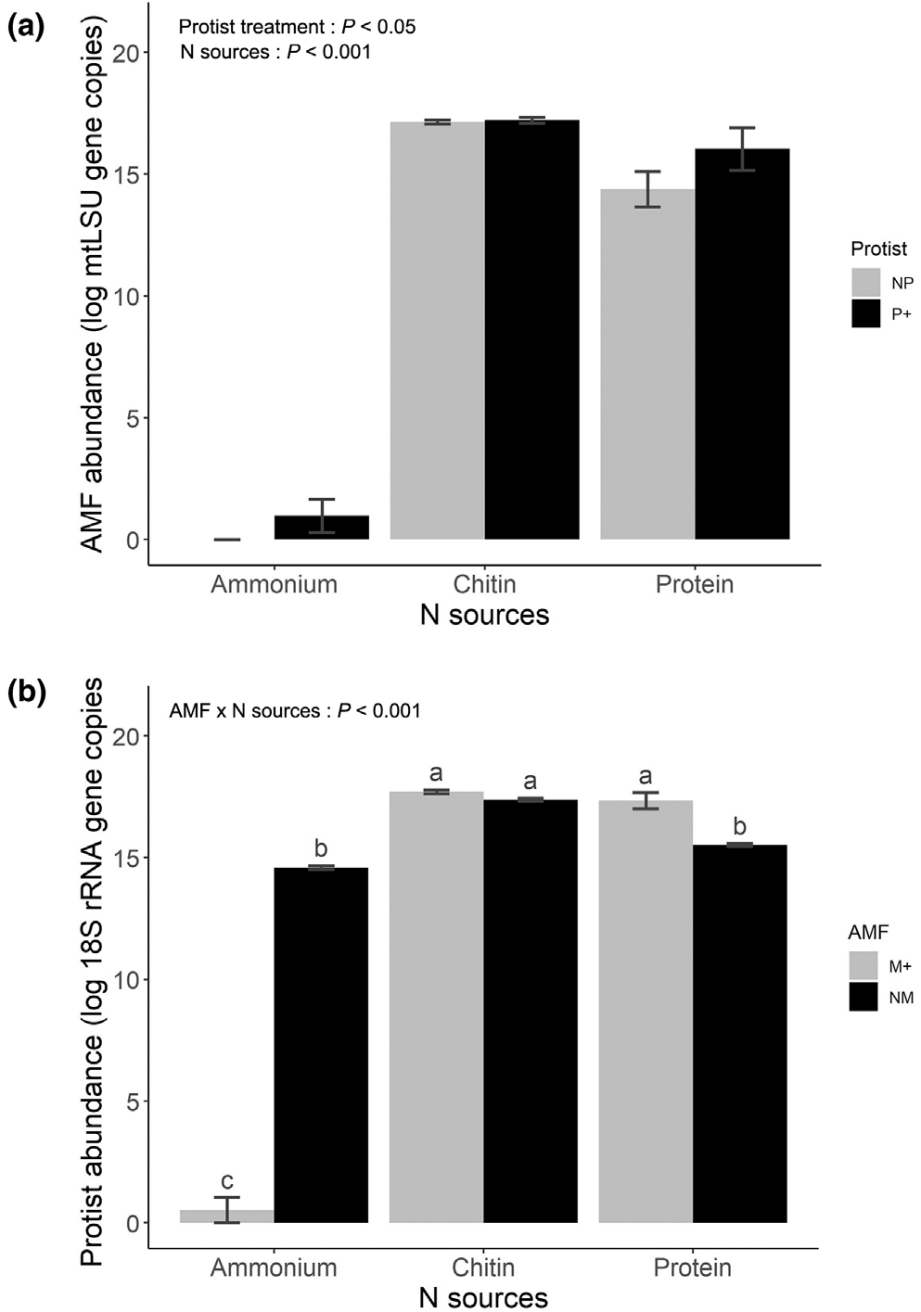
Abundance of arbuscular mycorrhizal fungus (AMF) *Rhizophagus irregularis* LPA9 (a) and the protist *Polysphondylium pallidum* (b) in the three hyphosphere compartments as assessed by quantitative real‐time PCR (qPCR). The qPCR primers targeted the mitochondrial large ribosomal subunit of AMF and 18S rRNA gene of protist. The analyses were conducted only for those microcosms wherein the respective organisms had been inoculated. Data were log‐transformed before analysis. A linear mixed‐effects analysis of variance (ANOVA) model with microcosm identity as random effect was employed to acknowledge potential codependency of analyses of three root‐free compartments from the same microcosm. The results are presented as mean ± SE (*n* = 21). Different lower‐case letters on individual bars indicate a statistically significant difference between the treatment means, as determined by Tukey's *post hoc* test (*P* < 0.05) following a significant two‐way ANOVA. M+, mycorrhizal treatment; NM, non‐mycorrhizal treatment; P+, protists present; NP, protists absent. Significance of the individual experimental factors, that is the presence of protists and identity of nitrogen (N) sources, is provided for (a), and significance of the three‐way interaction is provided for (b).

Arbuscular mycorrhizal fungus abundance in the hyphosphere compartments was significantly affected by N source identity (*F*_2,78_ = 573, *P* < 0.001) and was also slightly and positively affected by the presence of protists (*F*_1,39_ = 4.19, *P* = 0.04) across the different N sources, but not by their interaction (Table S1). The highest abundance of AMF was observed on chitin, followed by protein (not significantly different), and the lowest AMF abundance (on average) was recorded on NH_4_Cl. At the outset of the experiment, AMF rapidly colonized the NH_4_Cl compartment with finely branched hyphae (Fig. S3b). By the end of the experiment, however, hyphal abundance measured by qPCR reached its lowest level in the same compartment, being below detection limit in the absence of protists (Fig. 5a).

Protist abundance was significantly influenced by the interaction between AMF and the identity of the N sources (*F*_2,78_ = 596, *P* < 0.001). The presence of AMF increased the abundance of protists on protein (8.2 × 10^7^ ± 2.3 × 10^7^ 18S gene copies per root‐free compartment), while the values were lower in the absence of AMF (5.7 × 10^6^ ± 4.0 × 10^5^ 18S gene copies per root‐free compartment). Protist abundance on chitin was not significantly different from that on protein in the presence of AMF (Fig. 5b). The pattern of protist abundance on NH_4_Cl generally followed that observed for bacteria, with very low values detected in mycorrhizal compared with NM microcosms (Fig. 5b, please note the logarithmic scale).

## Discussion

### Roots access to N in the AMF hyphosphere

The increase in ^15^N in the mycorrhizal roots is a noteworthy finding, as it indicates a genuine transfer of N from the distant root‐free compartments to the roots via the AMF hyphae. This finding corroborates previous research indicating that AMF are capable of exploiting N patches at a considerable distance from roots and transporting such resources back to the roots (Govindarajulu *et al*., 2005; Leigh *et al*., 2009; Thirkell *et al*., 2016). It also fits well with the previous reports on the AMF hyphae playing an active role in the movement of N from organic amendments to the plants (Hodge *et al*., 2001; Koller *et al*., 2013; Bukovská *et al*., 2018; Rozmoš *et al*., 2022; Duan *et al*., 2024). Furthermore, a low but quantifiable transfer of ^15^N to roots from the ammonium or chitin sources was observed in NM microcosms. Our experimental design effectively impeded the transfer of soluble compounds from the hyphosphere compartments to the roots. Nevertheless, the ^15^N transfer may have been due to the diffusion of NH_3_ gas, which might be spontaneously released from the ammonium and chitin compartments (N from the latter being previously mineralized by the bacteria) and presented in the lateral N flow pathway (Hestrin *et al*., 2021).

Seven days after the addition of the different N sources into the hyphosphere compartments, the AMF hyphae proliferated vigorously on NH_4_Cl as compared to in the chitin and protein compartments (details not shown). Furthermore, the AMF hyphae took up large quantities of ^15^N from labeled NH_4_Cl and transported it toward the roots, and this was accompanied by reduced abundances of bacteria and protists in the same compartment. These findings are consistent with our previous report (Rozmoš *et al*., 2022) and other available literature (Tanaka & Yano, 2005; Xu *et al*., 2018; Savolainen & Kytöviita, 2022), describing competition for easily available N between the AMF hyphae and other microbes and/or plant roots.

### AMF depend on microbial interplay in the hyphosphere for organic N uptake

We hypothesized that bacteria and protists would facilitate the uptake of organic N by hyphae into roots. Because of their limited exo‐enzymatic activity, it is unlikely that AMF can degrade organic compounds independently. This has been supported by experimental evidence (Jansa *et al*., 2018; Rozmoš *et al*., 2022; Duan *et al*., 2024). Our findings align with the first hypothesis, indicating that the uptake of ^15^N by the AMF hyphae from the chitin and protein sources was facilitated by bacteria and protists, respectively. This finding supports the theory that the chitinolytic activity of *P. chitinolyticus* CCM4527 effectively facilitated the mineralization of ^15^N in the chitin compartment. Breakdown of chitin results in the formation of acetate, glucose, and ammonium ions, all of which serve as easily available sources of C and N for microbes (Blank & Hinman, 2016; Ma *et al*., 2020). Subsequently, the N (including the ^15^N) after chitin mineralization was taken up by the AMF hyphae and transferred into the roots. Similarly, Rozmoš *et al*. (2022) reported that AMF require the assistance of chitinolytic bacteria to acquire N from chitin in the hyphosphere.

The transfer of ^15^N from the protein source to plants was minimal in the absence of protists, despite the presence of bacteria possessing proteolytic activity. It seems that the rate of protein depolymerization by *P. chitinolyticus* was slow, as also indicated in a plate assay (Fig. S1). This rate is likely insufficient to meet the requirements of AMF, which in turn are unable to facilitate the transfer of significant amounts of N from proteins to the roots. Research indicates that when N is scarce, AMF utilize a significant portion of N for forming their own tissue (Hodge & Fitter, 2010) and only the excess N is then translocated to the host plant (Hodge *et al*., 2001; Leigh *et al*., 2011; Farrell *et al*., 2014). This is corroborated by the low ratio of ^15^N in the roots to ^15^N in the hyphae (Fig. 3). Once protists were introduced into the hyphosphere compartment with proteins, the mobilization of ^15^N from the protein source was markedly enhanced, accompanied by much increased ^15^N allocation to both roots and hyphae (Fig. 2). These results suggest that protists probably engulf protein clumps directly and mineralize such N in the AMF hyphosphere. This concept is corroborated by the findings of Thao *et al*. (2014), who demonstrated that protists were capable of degrading complete proteins from seawater even as bacterial cells were inactivated and unable to produce proteases. It is also possible that protein N is mineralized through the so‐called microbial loop, whereby N immobilized primarily in the bacterial biomass is effectively mobilized through protists' grazing and subsequent ammonium excretion (Clarholm, 1985; Bonkowski, 2004; Bonkowski & Clarholm, 2012; Bukovská *et al*., 2018). Although our findings generally support the first hypotheses, we cannot unequivocally state what exactly happened in the protein‐enriched hyphosphere, as we did not include protein‐enriched root‐free compartments with protists and without bacteria into our experimental design (Fig. S2). In any case, protists proved instrumental for the release of N from proteins in the AMF hyphosphere to levels unmatched by bacteria alone.

### AMF regulates bacterial and protist abundance in the hyphosphere compartments

Contrary to our second hypothesis that AMF would enhance the abundance of bacteria and protists, a decrease in bacterial abundance was observed in the chitin compartment in the presence of AMF while their abundance remained unaffected by AMF in the protein compartment (Fig. 4). Conversely, protist abundance remained unaffected by AMF in the chitin compartment while it increased in the protein compartment in the presence of AMF. These findings compel us to reject our second hypothesis and indicate that AMF do not invariably induce their hyphal companion microbes but rather regulate their abundance according to the situation. We observed that in the presence of AMF, for instance, the abundance of bacteria remained stable alongside protists (Fig. 4). Conversely, in the absence of mycorrhiza, a clear negative interaction was observed, characterized by lower bacterial abundance when protists were present (Fig. 4). These findings align with the two key mechanisms for controlling trophic structure, where protists exert a top‐down control over bacterial populations in the rhizosphere (Kreuzer *et al*., 2006; Rosenberg *et al*., 2009) while AMF modify resource availability from the bottom‐up by competing for mineral nutrients while providing hyphal exudate as a C source in the hyphosphere (Marschner & Baumann, 2003; Rillig *et al*., 2006; Rodríguez‐Caballero *et al*., 2017; Hünninghaus *et al*., 2019). Arbuscular mycorrhizal fungi demonstrated themselves to be highly competitive with both bacteria and protists in acquiring inorganic N, leading to low abundances of the latter microbes on NH_4_Cl (Figs 4, 5). It can be suggested that the AMF hyphae had largely exhausted the available NH_4_^+^ ions before protists and bacteria entered the compartment, resulting in nutrient‐limiting conditions for both latter organisms with respect to N. Previous studies have also shown that AMF may compete with bacterial and fungal communities by reducing NH_4_^+^ concentration in the soil solution (Bukovská *et al*., 2018; Veresoglou *et al*., 2019; Dudáš *et al*., 2022). Nutrient‐limiting conditions may thus force the chitinolytic bacteria to become opportunistic inhabitants on the AMF hyphae to meet their nutrient requirements (Jansa *et al*., 2020). This phenomenon of bacteria feeding on the AMF hyphae could possibly explain the observed disappearance of the mycorrhizal fungal DNA on NH_4_Cl in the later stage of the experiment (Fig. 5a). An alternative explanation for that disappearance would be retraction of the AMF protoplasm, including nuclei and mitochondria, from the NH_4_Cl compartment at a later stage of the experiment due to a lack of available resources, which could be taken up while leaving the empty hyphal walls with retraction septa behind. This phenomenon has been observed before (Pepe *et al*., 2018), and it would be consistent with our observation that hyphae were present in that compartment while failing to detect any mycorrhizal fungal DNA. (We did not aim to distinguish microscopically between living and dead hyphae.)

### Feedback effects of protists on AMF in the hyphosphere

A pronounced impact of protists on N recycling was observed in the hyphosphere amended with proteins. The abundance of AMF increased slightly in the presence of protists within the protein compartment, and this was associated with a more efficient transfer of ^15^N to the roots. These findings suggest that protists not only assist in the mineralization of N from proteins but also promote the growth of the AMF hyphae. Inasmuch as there was no easily available source of C in the hyphosphere compartment (except proteins, which would have to be depolymerized first), but easily available C was accessible in ample amounts outside of the hyphosphere compartments, it can be hypothesized that, when protists are present, the extraradical AMF hyphae exude more C in exchange for mobilized N, thereby facilitating microbial growth in the hyphosphere (Johnson *et al*., 2010; Fellbaum *et al*., 2012; Koller *et al*., 2013). Although the experiment reported here was conducted using root organ cultures without any shoots, and it was not possible unequivocally to identify the origin of C for protists and/or bacteria due to an absence of isotopic C labeling, the observations of lower biomass and lower C content in mycorrhizal roots compared with NM roots indirectly support the hypothesis stated previously, indicating that AMF (and their associated microbes) used as much as 20–25% of the easily available C in the microcosms (Notes S1; Figs S5, S6). By contrast, the N content of roots was not at all affected by the presence of AMF or protists (Notes S1; Fig. S7).

### Conclusion and future perspectives

**Fig. 6:**
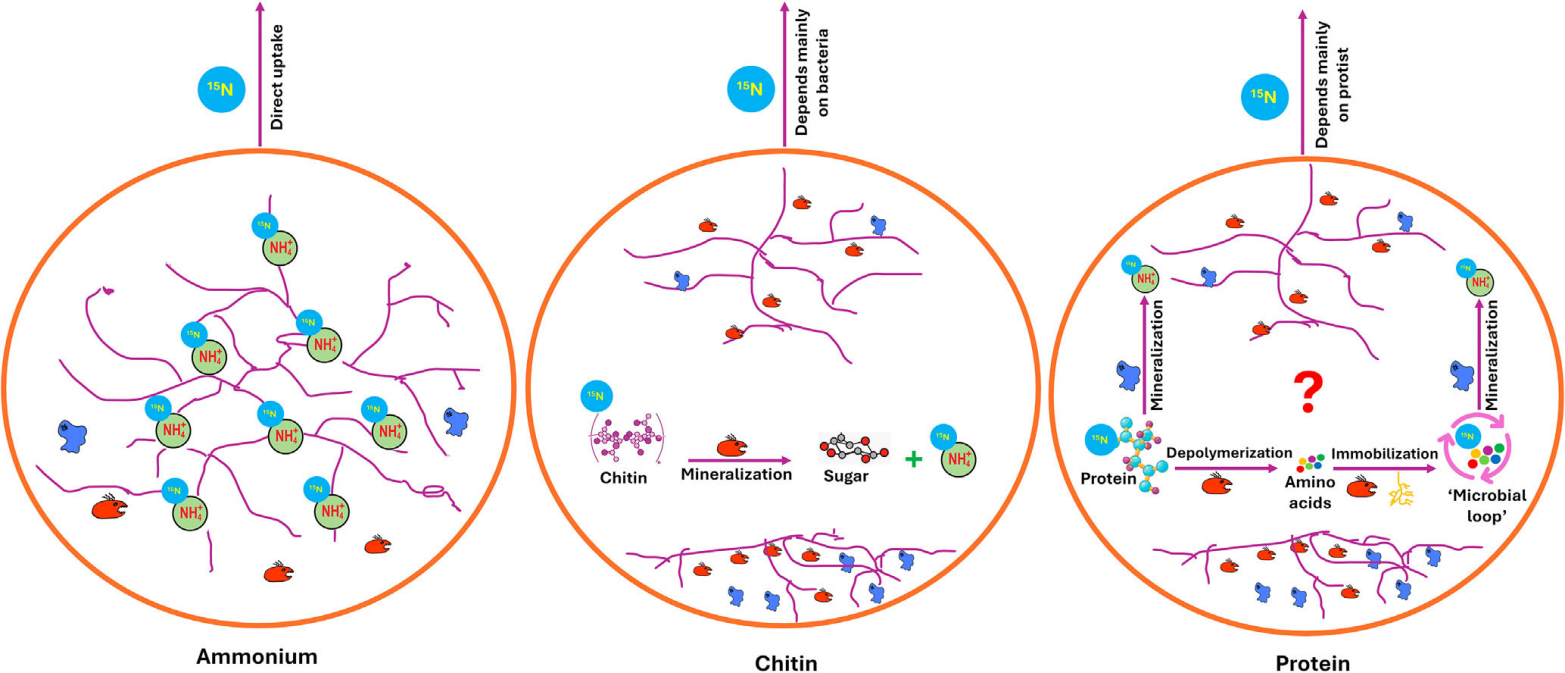
Overview of organic nitrogen (N) mineralization in the hyphosphere compartments and the mycorrhiza‐mediated uptake of N from those compartments. When using ammonium (NH_4_Cl) as a source of inorganic N, the mycorrhizal fungus showed itself to be in competition for the resource with bacteria and protists. The fungus can take up ammonium ions (NH_4_^+^) directly and transfer N to the roots, resulting in effective transport of ^15^N from labeled NH_4_Cl to roots under all microbial scenarios. In the case of chitin as an N source, an effect of chitinolytic bacteria (shown in red) was observed. Here, ^15^N released from chitin is taken up by mycorrhizal hyphae (shown in purple) and subsequently transferred to the roots and is not really much affected by the presence of protists (shown in blue). High levels of ^15^N transport from labeled chitin into nonmycorrhizal roots are consistent with efficient (bacteria‐driven) ammonification and subsequent ammonia volatilization. The end products of chitin degradation, such as sugar monomers and ammonium ions, support the growth of all microbes in the hyphosphere compartment. With protein as the N source, a priming effect of arbuscular mycorrhizal fungi was observed, during which hyphal exudates regulate the growth of bacteria and protists. This enhances the ability of protists to mineralize ^15^N from protein, which is then taken up by hyphae and transferred into the roots. The bacteria thrive better in the presence of mycorrhizal hyphae and protists than in absence of mycorrhizal hyphae but the presence of protists. Likewise, protists thrive better in the presence of mycorrhizal hyphae than in their absence.

The compartmented microcosm setup used in this study allowed us to compare AMF hyphae's N uptake efficiency from chitin and protein sources against NH_4_Cl. Our findings specifically addressed how the AMF hyphae interacted with hyphosphere microbes to facilitate organic N uptake. We found that the ^15^N from NH_4_Cl was directly absorbed by the AMF hyphae. By contrast, the transfer of ^15^N from chitin and proteins primarily depended on chitinolytic bacteria and protists, respectively (Fig. 6). Although our experimental design did not yield detailed insights into protein N mineralization by protists, future research should seek to understand this process in detail and independent of bacterial involvement. Additionally, future studies should focus on the temporal dynamics of each microorganism involved in multitrophic interactions during the recycling of organic nutrients. Investigating organic N uptake through the AMF symbiosis is essential for terrestrial ecosystems, where N often is the primary growth‐limiting element for plants.

## Competing interests

None declared.

## Author contributions

JJ and MR conceived the research. MR, PB, HH and MK conducted the experiment, took photographs and carried out subsequent sample analyses. MR conducted preliminary statistical analyses and designed some of the illustrations. AV conducted data analyses, finalized all the illustrations and wrote the first draft of the manuscript. JJ contributed to revisions of the manuscript texts, and all authors approved the final manuscript before submission. AV and MR contributed equally to this work.

## Disclaimer

The New Phytologist Foundation remains neutral with regard to jurisdictional claims in maps and in any institutional affiliations.

## Supporting information


**Fig. S1** Chitinolytic and proteolytic activities of *Paenibacillus chitinolyticus* CCM4527.
**Fig. S2** Complete timeline and design of the experiment.
**Fig. S3** Development of *Rhizophagus irregularis* LPA9 hyphae and spores.
**Fig. S4** Proliferation of *Polysphondylium pallidum* in the different nitrogen source compartments.
**Fig. S5** Impact of arbuscular mycorrhizal fungi and protist presence on chicory root biomass per microcosm.
**Fig. S6** Impact of arbuscular mycorrhizal fungi and protist presence on chicory root carbon content per microcosm.
**Fig. S7** Impact of arbuscular mycorrhizal fungi and protist presence on chicory root nitrogen content per microcosm.
**Notes S1** Dry biomass, total carbon, and nitrogen contents of roots.
**Table S1** Results of analyses of variance for multiple variables tested in this study.

**Table S2** Primary experimental data.
Please note: Wiley is not responsible for the content or functionality of any Supporting Information supplied by the authors. Any queries (other than missing material) should be directed to the *New Phytologist* Central Office.

## Data Availability

The primary experimental data that support the findings presented in this paper are all available in the Table S2 accompanying this article.

## References

[nph70153-bib-0001] Adamczyk B . 2021. How do terrestrial plants access high molecular mass organic nitrogen, and why does it matter for soil organic matter stabilization? Plant and Soil 465: 583–592.

[nph70153-bib-0002] Blank CE , Hinman NW . 2016. Cyanobacterial and algal growth on chitin as a source of nitrogen; ecological, evolutionary, and biotechnological implications. Algal Research 15: 152–163.

[nph70153-bib-0003] Bonkowski M . 2004. Protozoa and plant growth: the microbial loop in soil revisited. New Phytologist 162: 617–631. 33873756 10.1111/j.1469-8137.2004.01066.x

[nph70153-bib-0004] Bonkowski M , Clarholm M . 2012. Stimulation of plant growth through interactions of bacteria and protozoa: testing the auxiliary microbial loop hypothesis. Acta Protozoologica 51: 237–247.

[nph70153-bib-0005] Bukovská P , Bonkowski M , Konvalinková T , Beskid O , Hujslová M , Püschel D , Řezáčová V , Gutiérrez‐Núñez MS , Gryndler M , Jansa J . 2018. Utilization of organic nitrogen by arbuscular mycorrhizal fungi—is there a specific role for protists and ammonia oxidizers? Mycorrhiza 28: 269–283. 29455336 10.1007/s00572-018-0825-0

[nph70153-bib-0006] Bukovská P , Püschel D , Hršelová H , Jansa J , Gryndler M . 2016. Can inoculation with living soil standardize microbial communities in soilless potting substrates? Applied Soil Ecology 108: 278–287.

[nph70153-bib-0007] Clarholm M . 1985. Interactions of bacteria, protozoa and plants leading to mineralization of soil nitrogen. Soil Biology and Biochemistry 17: 181–187.

[nph70153-bib-0008] Coskun D , Britto D , Shi W , Kronzucker HJ . 2017. Nitrogen transformations in modern agriculture and the role of biological nitrification inhibition. Nature Plants 3: 17074. 28585561 10.1038/nplants.2017.74

[nph70153-bib-0009] Cranenbrouck S , Voets L , Bivort C , Renard L , Strullu DG , Declerck S . 2005. Methodologies for *in vitro* cultivation of arbuscular mycorrhizal fungi with root organs. In: Declerck S , Fortin JA , Strullu DG , eds. *In vitro* culture of mycorrhizas. Soil biology, vol. 4. Berlin, Heidelberg: Springer, 341–375.

[nph70153-bib-0010] Czaban W , Jämtgård S , Näsholm T , Rasmussen J , Nicolaisen M , Fomsgaard IS . 2016. Direct acquisition of organic N by white clover even in the presence of inorganic N. Plant and Soil 407: 91–107.

[nph70153-bib-0011] Duan S , Feng G , Limpens E , Bonfante P , Xie X , Zhang L . 2024. Cross‐kingdom nutrient exchange in the plant–arbuscular mycorrhizal fungus–bacterium continuum. Nature Reviews Microbiology 22: 773–790. 39014094 10.1038/s41579-024-01073-7

[nph70153-bib-0012] Dudáš M , Pjevac P , Kotianová M , Gančarčíková K , Rozmoš M , Hršelová H , Bukovská P , Jansa J . 2022. Arbuscular mycorrhiza and nitrification: disentangling processes and players through using synthetic nitrification inhibitors. Applied and Environmental Microbiology 88: e01369‐22. 36190238 10.1128/aem.01369-22 PMC9599619

[nph70153-bib-0013] Enggrob KL , Jakobsen CM , Pedersen IF , Rasmussen J . 2019. Newly depolymerized large organic N contributes directly to amino acid uptake in young maize plants. New Phytologist 224: 689–699. 31325391 10.1111/nph.16070

[nph70153-bib-0014] Farrell M , Prendergast‐Miller M , Jones DL , Hill PW , Condron LM . 2014. Soil microbial organic nitrogen uptake is regulated by carbon availability. Soil Biology and Biochemistry 77: 261–267.

[nph70153-bib-0015] Farzadfar S , Knight JD , Congreves KA . 2021. Soil organic nitrogen: an overlooked but potentially significant contribution to crop nutrition. Plant and Soil 462: 7–23. 34720208 10.1007/s11104-021-04860-w PMC8550315

[nph70153-bib-0016] Fellbaum CR , Gachomo EW , Beesetty Y , Choudhari S , Strahan GD , Pfeffer PE , Kiers ET , Bücking H . 2012. Carbon availability triggers fungal nitrogen uptake and transport in arbuscular mycorrhizal symbiosis. Proceedings of the National Academy of Sciences, USA 109: 2666–2671. 10.1073/pnas.1118650109 PMC3289346 22308426

[nph70153-bib-0017] Govindarajulu M , Pfeffer P , Jin H , Abubaker J , Douds DD , Allen JW , Bücking H , Lammers PJ , Shachar‐Hill Y . 2005. Nitrogen transfer in the arbuscular mycorrhizal symbiosis. Nature 435: 819–823. 15944705 10.1038/nature03610

[nph70153-bib-0018] Grandy AS , Daly AB , Bowles TM , Gaudin AC , Jilling A , Leptin A , McDaniel MD , Wade J , Waterhouse H . 2022. The nitrogen gap in soil health concepts and fertility measurements. Soil Biology and Biochemistry 175: 108856.

[nph70153-bib-0019] Gryndler M , Trilčová J , Hršelová H , Streiblová E , Gryndlerová H , Jansa J . 2013. *Tuber aestivum* Vittad. mycelium quantified: advantages and limitations of a qPCR approach. Mycorrhiza 23: 341–348. 23271632 10.1007/s00572-012-0475-6

[nph70153-bib-0020] Hagh‐Doust N , Mikryukov V , Anslan S , Bahram M , Puusepp R , Dulya O , Tedersoo L . 2023. Effects of nitrogen deposition on carbon and nutrient cycling along a natural soil acidity gradient as revealed by metagenomics. New Phytologist 238: 2607–2620. 36949609 10.1111/nph.18897

[nph70153-bib-0021] Henkes GJ , Kandeler E , Marhan S , Scheu S , Bonkowski M . 2018. Interactions of mycorrhiza and protists in the rhizosphere systemically alter microbial community composition, plant shoot‐to‐root ratio and within‐root system nitrogen allocation. Frontiers in Environmental Science 6: 117.

[nph70153-bib-0022] Herdler S , Kreuzer K , Scheu S , Bonkowski M . 2008. Interactions between arbuscular mycorrhizal fungi (*Glomus intraradices*, Glomeromycota) and amoebae (*Acanthamoeba castellanii*, Protozoa) in the rhizosphere of rice (*Oryza sativa*). Soil Biology and Biochemistry 40: 660–668.

[nph70153-bib-0023] Hestrin R , Hammer EC , Mueller CW , Lehmann J . 2019. Synergies between mycorrhizal fungi and soil microbial communities increase plant nitrogen acquisition. Communications Biology 2: 233. 31263777 10.1038/s42003-019-0481-8 PMC6588552

[nph70153-bib-0024] Hestrin R , Weber PK , Pett‐Ridge J , Lehmann J . 2021. Plants and mycorrhizal symbionts acquire substantial soil nitrogen from gaseous ammonia transport. New Phytologist 231: 1746–1757. 34077566 10.1111/nph.17527

[nph70153-bib-0025] Hodge A , Campbell CD , Fitter AH . 2001. An arbuscular mycorrhizal fungus accelerates decomposition and acquires nitrogen directly from organic material. Nature 413: 297–299. 11565029 10.1038/35095041

[nph70153-bib-0026] Hodge A , Fitter AH . 2010. Substantial nitrogen acquisition by arbuscular mycorrhizal fungi from organic material has implications for N cycling. Proceedings of the National Academy of Sciences, USA 107: 13754–13759. 10.1073/pnas.1005874107 PMC2922220 20631302

[nph70153-bib-0027] Holz M , Lewin S , Kolb S , Becker JN , Bergmann J . 2024. How to get to the N – a call for interdisciplinary research on organic N utilization pathways by plants. Plant and Soil 508: 955–969.

[nph70153-bib-0028] Hoysted GA , Field KJ , Sinanaj B , Bell CA , Bidartondo MI , Pressel S . 2023. Direct nitrogen, phosphorus and carbon exchanges between *Mucoromycotina* ‘fine root endophyte’ fungi and a flowering plant in novel monoxenic cultures. New Phytologist 238: 70–79. 36739554 10.1111/nph.18630 PMC10952891

[nph70153-bib-0029] Hünninghaus M , Dibbern D , Kramer S , Koller R , Pausch J , Schloter‐Hai B , Lueders T . 2019. Disentangling carbon flow across microbial kingdoms in the rhizosphere of maize. Soil Biology and Biochemistry 134: 122–130.

[nph70153-bib-0030] Jämtgård S , Näsholm T , Huss‐Danell K . 2008. Characteristics of amino acid uptake in barley. Plant and Soil 302: 221–231.

[nph70153-bib-0031] Jansa J , Bukovská P , Hršelová H , Püschel D . 2018. Utilization of organic nitrogen by arbuscular mycorrhizal hyphae in soil‐zooming into the hyphosphere microbiome. Journal of Integrated Field Science 15: 2–7.

[nph70153-bib-0032] Jansa J , Šmilauer P , Borovička J , Hršelová H , Forczek ST , Slámová K , Řezanka T , Rozmoš M , Bukovská P , Gryndler M . 2020. Dead *Rhizophagus irregularis* biomass mysteriously stimulates plant growth. Mycorrhiza 30: 63–77. 32062707 10.1007/s00572-020-00937-z

[nph70153-bib-0033] Johnson NC , Wilson GW , Bowker MA , Wilson JA , Miller RM . 2010. Resource limitation is a driver of local adaptation in mycorrhizal symbioses. Proceedings of the National Academy of Sciences, USA 107: 2093–2098. 10.1073/pnas.0906710107 PMC2836645 20133855

[nph70153-bib-0034] Koller R , Rodriguez A , Robin C , Scheu S , Bonkowski M . 2013. Protozoa enhance foraging efficiency of arbuscular mycorrhizal fungi for mineral nitrogen from organic matter in soil to the benefit of host plants. New Phytologist 199: 203–211. 23534902 10.1111/nph.12249

[nph70153-bib-0035] Kreuzer K , Adamczyk J , Iijima M , Wagner M , Scheu S , Bonkowski M . 2006. Grazing of a common species of soil protozoa (*Acanthamoeba castellanii*) affects rhizosphere bacterial community composition and root architecture of rice (*Oryza sativa* L.). Soil Biology and Biochemistry 38: 1665–1672.

[nph70153-bib-0036] Kuzyakov Y , Xu X . 2013. Competition between roots and microorganisms for nitrogen: mechanisms and ecological relevance. New Phytologist 198: 656–669. 23521345 10.1111/nph.12235

[nph70153-bib-0100] Kuroshima KI , Sakane T , Takata R , Yokota A . 1996. *Bacillus ehimensis* sp. nov. and *Bacillus chitinolyticus* sp. nov., new chitinolytic members of the genus *Bacillus* . International Journal of Systematic Bacteriology 46: 76–80.

[nph70153-bib-0037] Lassaletta L , Billen G , Grizzetti B , Anglade J , Garnier J . 2014. 50 year trends in nitrogen use efficiency of world cropping systems: the relationship between yield and nitrogen input to cropland. Environmental Research Letters 9: 105011.

[nph70153-bib-0101] Lee JS , Pyun YR , Bae KS . 2004. Transfer of *Bacillus ehimensis* and *Bacillus chitinolyticus* to the genus *Paenibacillus* with emended descriptions of *Paenibacillus ehimensis* comb. nov. and *Paenibacillus chitinolyticus* comb. nov. International Journal of Systematic and Evolutionary Microbiology 54: 929–933. 15143044 10.1099/ijs.0.02765-0

[nph70153-bib-0038] Leigh J , Fitter AH , Hodge A . 2011. Growth and symbiotic effectiveness of an arbuscular mycorrhizal fungus in organic matter in competition with soil bacteria. FEMS Microbiology Ecology 76: 428–438. 21303398 10.1111/j.1574-6941.2011.01066.x

[nph70153-bib-0039] Leigh J , Hodge A , Fitter AH . 2009. Arbuscular mycorrhizal fungi can transfer substantial amounts of nitrogen to their host plant from organic material. New Phytologist 181: 199–207. 18811615 10.1111/j.1469-8137.2008.02630.x

[nph70153-bib-0040] Liu D , Huang Y , Yan H , Jiang Y , Zhao T , An S . 2018. Dynamics of soil nitrogen fractions and their relationship with soil microbial communities in two forest species of northern China. PLoS ONE 13: e0196567. 29795562 10.1371/journal.pone.0196567 PMC5967799

[nph70153-bib-0041] Ma X , Gözaydın G , Yang H , Ning W , Han X , Poon NY , Liang H , Yan N , Zhou K . 2020. Upcycling chitin‐containing waste into organonitrogen chemicals via an integrated process. Proceedings of the National Academy of Sciences, USA 117: 7719–7728. 10.1073/pnas.1919862117 PMC7149430 32213582

[nph70153-bib-0042] Marschner P , Baumann K . 2003. Changes in bacterial community structure induced by mycorrhizal colonisation in split‐root maize. Plant and Soil 251: 279–289.

[nph70153-bib-0043] Näsholm T , Kielland K , Ganeteg U . 2009. Uptake of organic nitrogen by plants. New Phytologist 182: 31–48. 19210725 10.1111/j.1469-8137.2008.02751.x

[nph70153-bib-0044] Niu L , Zhang H , Wu Z , Wang Y , Liu H , Wu X , Wang W . 2018. Modified TCA/acetone precipitation of plant proteins for proteomic analysis. PLoS ONE 13: e0202238. 30557402 10.1371/journal.pone.0202238 PMC6296544

[nph70153-bib-0045] Paungfoo‐Lonhienne C , Lonhienne TG , Rentsch D , Robinson N , Christie M , Webb RI , Gamage HK , Carroll BJ , Schenk PM , Schmidt S . 2008. Plants can use protein as a nitrogen source without assistance from other organisms. Proceedings of the National Academy of Sciences, USA 105: 4524–4529. 10.1073/pnas.0712078105 PMC2393761 18334638

[nph70153-bib-0046] Pepe A , Giovannetti M , Sbrana C . 2018. Lifespan and functionality of mycorrhizal fungal mycelium are uncoupled from host plant lifespan. Scientific Reports 8: 10235. 29980700 10.1038/s41598-018-28354-5 PMC6035242

[nph70153-bib-0047] Phillips DL , Gregg JW . 2001. Uncertainty in source partitioning using stable isotopes. Oecologia 127: 171–179. 24577646 10.1007/s004420000578

[nph70153-bib-0048] Pinheiro J , Bates D , R Core Team . 2023. nlme: linear and nonlinearmixed effects models . R package v.3. 1‐164.

[nph70153-bib-0049] Püschel D , Janoušková M , Hujslová M , Slavíková R , Gryndlerová H , Jansa J . 2016. Plant–fungus competition for nitrogen erases mycorrhizal growth benefits of *Andropogon gerardii* under limited nitrogen supply. Ecology and Evolution 6: 4332–4346. 27386079 10.1002/ece3.2207 PMC4930984

[nph70153-bib-0050] R Development Core Team . 2024. R: a language and environment for statistical computing, v.4.4.2. Vienna, Austria: R Foundation for Statistical Computing. http://www.r‐project.org.

[nph70153-bib-0051] Rillig MC , Mummey DL , Ramsey PW , Klironomos JN , Gannon JE . 2006. Phylogeny of arbuscular mycorrhizal fungi predicts community composition of symbiosis‐associated bacteria. FEMS Microbiology Ecology 57: 389–395. 16907753 10.1111/j.1574-6941.2006.00129.x

[nph70153-bib-0052] Rodríguez‐Caballero G , Caravaca F , Fernández‐González AJ , Alguacil MM , Fernández‐López M , Roldán A . 2017. Arbuscular mycorrhizal fungi inoculation mediated changes in rhizosphere bacterial community structure while promoting revegetation in a semiarid ecosystem. Science of the Total Environment 584: 838–848. 28131451 10.1016/j.scitotenv.2017.01.128

[nph70153-bib-0053] Rosenberg K , Bertaux J , Krome K , Hartmann A , Scheu S , Bonkowski M . 2009. Soil amoebae rapidly change bacterial community composition in the rhizosphere of *Arabidopsis thaliana* . The ISME Journal 3: 675–684. 19242534 10.1038/ismej.2009.11

[nph70153-bib-0054] Rozmoš M , Bukovská P , Hršelová H , Kotianová M , Dudáš M , Gančarčíková K , Jansa J . 2022. Organic nitrogen utilisation by an arbuscular mycorrhizal fungus is mediated by specific soil bacteria and a protist. The ISME Journal 16: 676–685. 34545172 10.1038/s41396-021-01112-8 PMC8857242

[nph70153-bib-0055] Savolainen T , Kytöviita MM . 2022. Mycorrhizal symbiosis changes host nitrogen source use. Plant and Soil 471: 643–654.

[nph70153-bib-0056] Tanaka Y , Yano K . 2005. Nitrogen delivery to maize via mycorrhizal hyphae depends on the form of N supplied. Plant, Cell & Environment 28: 1247–1254.

[nph70153-bib-0057] Tegeder M , Masclaux‐Daubresse C . 2018. Source and sink mechanisms of nitrogen transport and use. New Phytologist 217: 35–53. 29120059 10.1111/nph.14876

[nph70153-bib-0058] Thao NV , Obayashi Y , Yokokawa T , Suzuki S . 2014. Coexisting protist‐bacterial community accelerates protein transformation in microcosm experiments. Frontiers in Marine Science 1: 69.

[nph70153-bib-0059] Thirkell TJ , Cameron DD , Hodge A . 2016. Resolving the ‘nitrogen paradox’ of arbuscular mycorrhizas: fertilization with organic matter brings considerable benefits for plant nutrition and growth. Plant, Cell & Environment 39: 1683–1690. 10.1111/pce.12667 PMC4982020 26510552

[nph70153-bib-0060] Thonar C , Erb A , Jansa J . 2012. Real‐time PCR to quantify composition of arbuscular mycorrhizal fungal communities‐‐marker design, verification, calibration and field validation. Molecular Ecology Resources 12: 219–232. 22059700 10.1111/j.1755-0998.2011.03086.x

[nph70153-bib-0061] Tisserant E , Malbreil M , Kuo A , Kohler A , Symeonidi A , Balestrini R , Charron P , Duensing N , Freidit Frey N , Gianinazzi‐Pearson V *et al*. 2013. Genome of an arbuscular mycorrhizal fungus provides insight into the oldest plant symbiosis. Proceedings of the National Academy of Sciences, USA 110: 20117–20122. 10.1073/pnas.1313452110 PMC3864322 24277808

[nph70153-bib-0062] Veresoglou SD , Verbruggen E , Makarova O , Mansour I , Sen R , Rillig MC . 2019. Arbuscular mycorrhizal fungi alter the community structure of ammonia oxidizers at high fertility via competition for soil NH4+. Microbial Ecology 78: 147–158. 30402724 10.1007/s00248-018-1281-2

[nph70153-bib-0063] Wang M , Pendall E , Fang C , Li B . 2018. A global perspective on agroecosystem nitrogen cycles after returning crop residue. Agriculture, Ecosystems & Environment 266: 49–54.

[nph70153-bib-0064] Xu YF , Chu XT , Zhang XH , Liu Q , Miao YJ , Sun YF . 2018. The forms of nitrogen source influence the interaction between *Elymus nutans* Griseb. and arbuscular mycorrhizal fungi. South African Journal of Botany 119: 37–44.

[nph70153-bib-0065] Zhang C , Geisen S , Berendsen RL , van der Heijden MG . 2024. Specialized protist communities on mycorrhizal fungal hyphae. Mycorrhiza 34: 517–524. 39249534 10.1007/s00572-024-01167-3 PMC11604758

