# Supplementary material for: Protists are key players in the utilization of protein nitrogen in the arbuscular mycorrhizal hyphosphere
Source: New Phytol. 2025 Apr 22;246(6):2753–64. doi: 10.1111/nph.70153 (PMC12095988; doi:10.1111/nph.70153)
Supplement: Supplementary file 1 — Fig. S1 Chitinolytic and proteolytic activities of Paenibacillus chitinolyticus CCM4527. Fig. S2 Complete timeline and design of the experiment. Fig. S3 Development of Rhizophagus irregularis LPA9 hyphae and spores. Fig. S4 Proliferation of Polysphondylium pallidum in the different nitrogen source compartments. Fig. S5 Impact of arbuscular mycorrhizal fungi and protist presence on chicory root biomass per microcosm. Fig. S6 Impact of arbuscular mycorrhizal fungi and protist presence on chicory root carbon content per microcosm. Fig. S7 Impact of arbuscular mycorrhizal fungi and protist presence on chicory root nitrogen content per microcosm. Notes S1 Dry biomass, total carbon, and nitrogen contents of roots. Table S1 Results of analyses of variance for multiple variables tested in this study. [file NPH-246-2753-s001.pdf]

New Phytologist Supporting Information

Article title: Protists are key players in utilization of protein nitrogen in the arbuscular mycorrhizal hyphosphere

Authors: Anukool Vaishnav#, Martin Rozmoš#, Michala Kotianová, Hana Hršelová, Petra Bukovská, Jan Jansa*

* Corresponding author (jansa@biomed.cas.cz)

# Authors who contributed equally, both first authors

Article acceptance date: 27 March 2025

The following Supporting Information is available for this article:

Supporting Information Notes S1 page 2

Supporting Information Figure S1 page 3

Supporting Information Figure S2 page 4

Supporting Information Figure S3 page 5

Supporting Information Figure S4 page 6

Supporting Information Figure S5 page 7

Supporting Information Figure S6 page 8

Supporting Information Figure S7 page 9

Supporting Information Table S1 page 10

Supporting Information References page 11

Supporting Information Notes S1

Dry biomass, total carbon (C) and nitrogen (N) contents of roots

The dry biomass of mycorrhizal roots was significantly lower than that of non-mycorrhizal (NM) roots ($F_{1,79} = 449$, $p < 0.001$), and this difference was not affected by the presence of protists (Supporting Information Fig. S5). Similarly, C content was significantly lower in mycorrhizal roots compared to NM roots ($F_{1,79} = 395$, $p < 0.001$). Again, this was not influenced by protists (Supporting Information Fig. S6). No significant difference was observed between the total N content of mycorrhizal and NM roots (Supporting Information Fig. S7). It is evident that mycorrhizal roots transfer significant amounts of C to the arbuscular mycorrhizal fungal hyphae, which probably release part of that to interact with the other organisms in the hyphosphere compartments and to receive N in exchange (Hoysted *et al.*, 2023; Read *et al.*, 2024), particularly as shown here for utilization of N from proteins.

Supporting Information Figure S1:

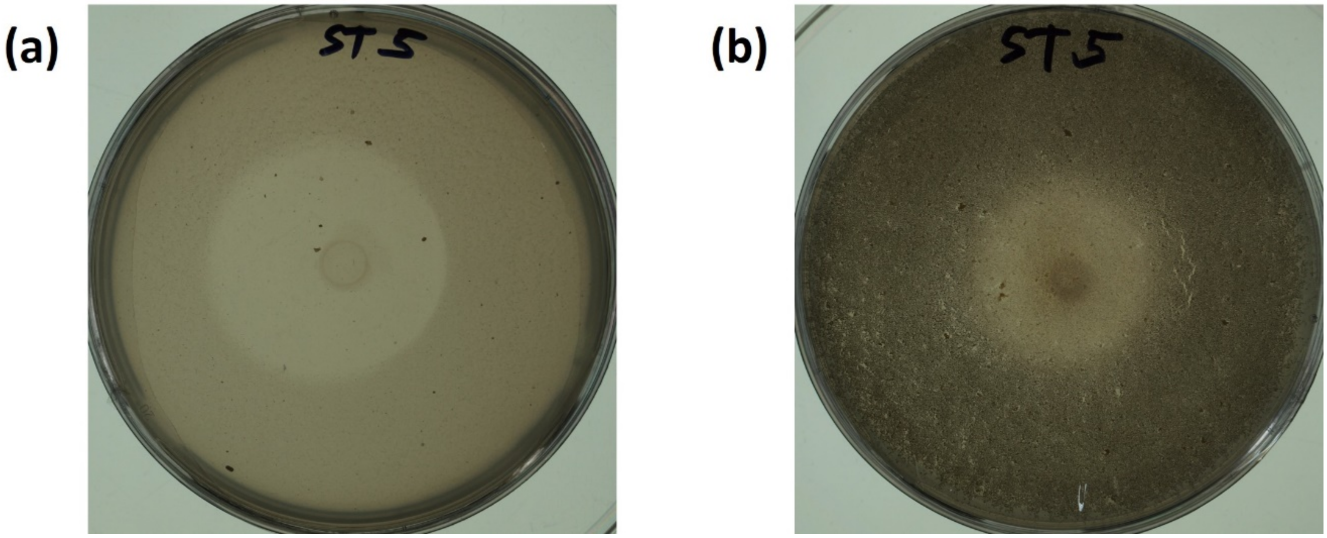

Chitinolytic and proteolytic activities of *Paenibacillus chitinolyticus* (Kuroshima et al., 1996) Lee et al., 2004 isolate CCM4527. Clearing zones are visible around the bacterial colonies on (a) colloidal chitin agar medium, and (b) casein agar medium. The experiment was conducted using standard Petri dishes (9 cm in diameter).

Supporting Information Figure S2:

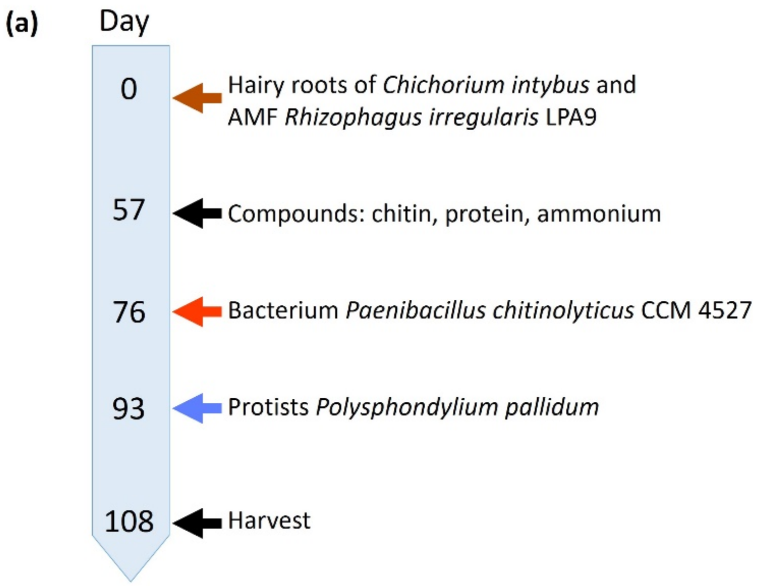

(b)

Mycorrhiza	Protist	Chitin	Protein	Ammonium
NM	protist +	^{15}N	^{14}N	^{14}N
NM	protist +	^{14}N	^{15}N	^{14}N
NM	protist +	^{14}N	^{14}N	^{15}N
NM	protist -	^{15}N	^{14}N	^{14}N
NM	protist -	^{14}N	^{15}N	^{14}N
NM	protist -	^{14}N	^{14}N	^{15}N
AMF	protist +	^{15}N	^{14}N	^{14}N
AMF	protist +	^{14}N	^{15}N	^{14}N
AMF	protist +	^{14}N	^{14}N	^{15}N
AMF	protist -	^{15}N	^{14}N	^{14}N
AMF	protist -	^{14}N	^{15}N	^{14}N
AMF	protist -	^{14}N	^{14}N	^{15}N

Complete timeline and design of the experiment. A complete day-wise timeline of the experiment (a) outlines all the events in chronological order. Design of the experiment (b) details the different treatments (each row) as combinations of three experimental factors: 1) mycorrhiza present (AMF), mycorrhiza absent (NM); 2) protists present (protist +), protists absent (protists -); and 3) isotopic labeling (^{15}N highlighted yellow) of the different nitrogen sources in each treatment.

.

Supporting Information Figure S3:

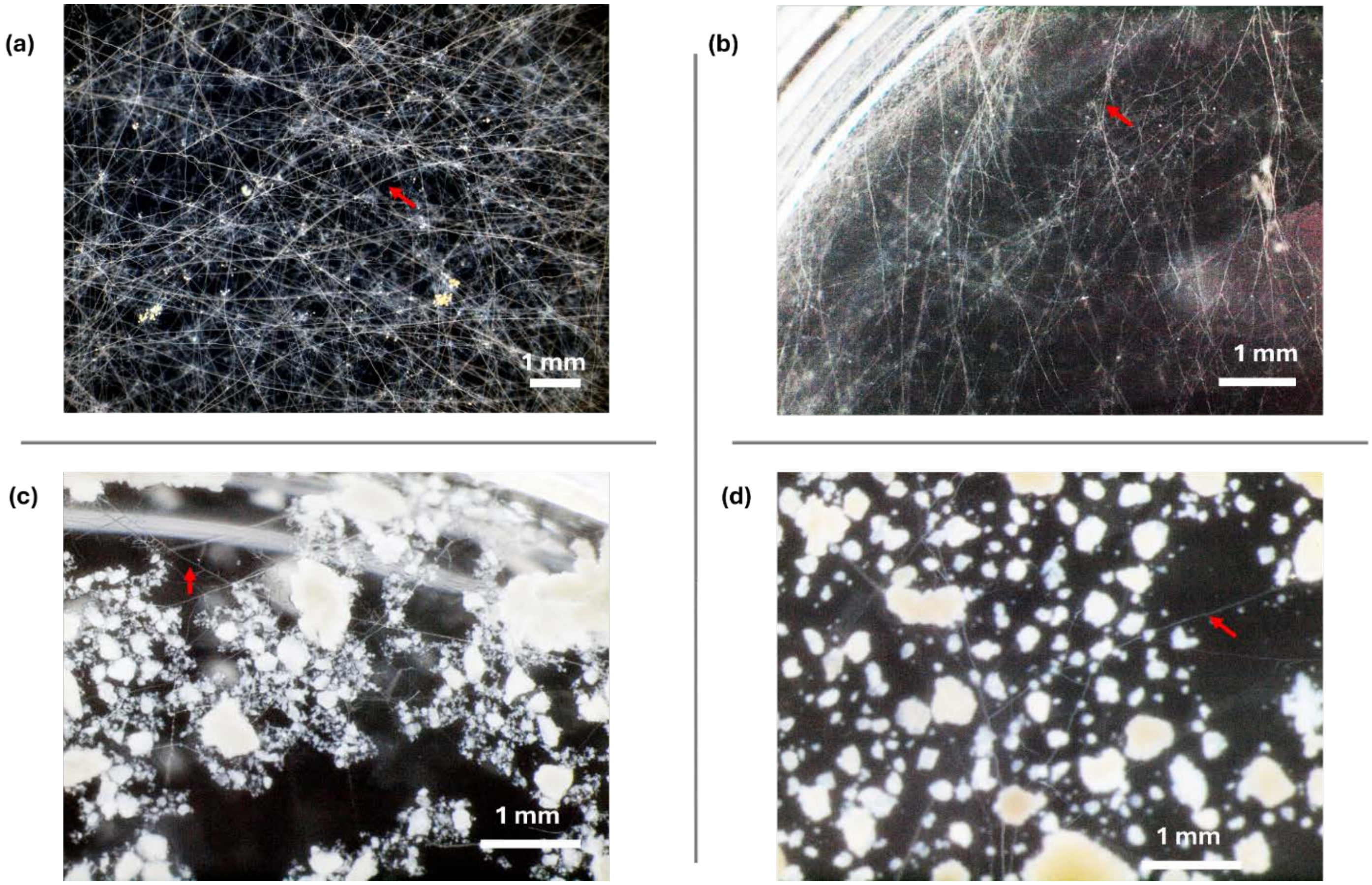

Development of *Rhizophagus irregularis* (Błaszk., Wubet, Renker & Buscot) C. Walker & A. Schüßler isolate LPA9 hyphae and spores. (a) outside the root compartment, (b) in the hyphosphere compartment amended with ammonium (NH_4Cl), (c) in the hyphosphere amended with chitin, and (d) in the hyphosphere amended with protein. The mycorrhizal fungal hyphae (red arrows) and spores developed in the main dish after they emerged from the root compartment. The photographs were taken on the 82nd day after setting up the experiment (i.e., 6 days after the bacterial inoculation) using an Olympus SZX10 stereomicroscope. The compartment amended with NH_4Cl exhibited long and extensively branched fungal hyphae compared to the other compartments.

Supporting Information Figure S4:

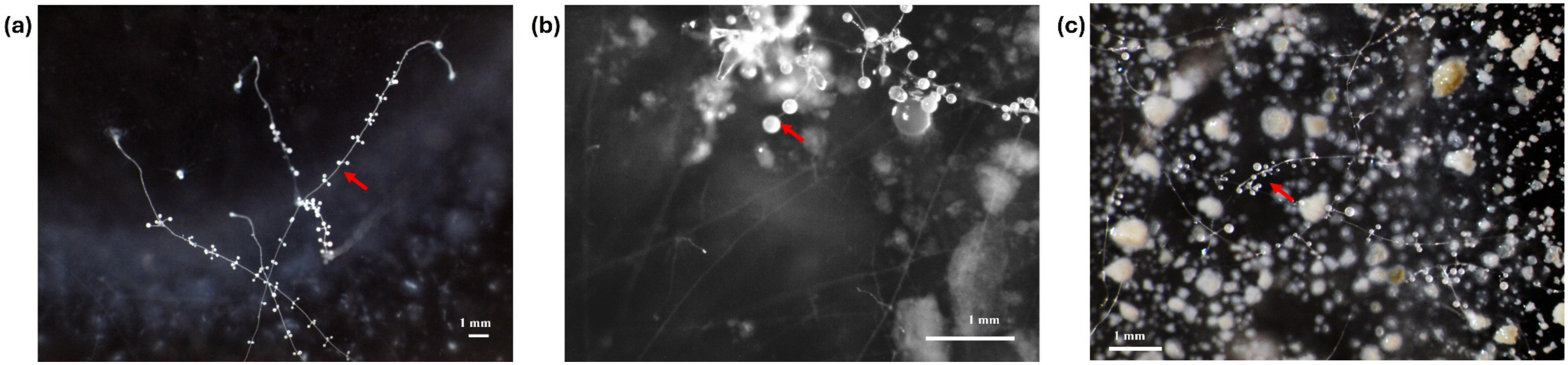

Proliferation of *Polysphondylium pallidum* Olive in the different nitrogen source compartments. The figure shows fruiting bodies in the different compartments as follow: (a) on ammonium (NH_4Cl), (b) on chitin, and (c) on protein. On the 93rd day of the experiment, the protist was inoculated into all three compartments. After 4 days of inoculation, the growth of the protist was observed under an Olympus SZX10 stereomicroscope. Mature fruiting bodies and whorled sorocarps were observed in all the compartments (red arrows).

Supporting Information Figure S5:

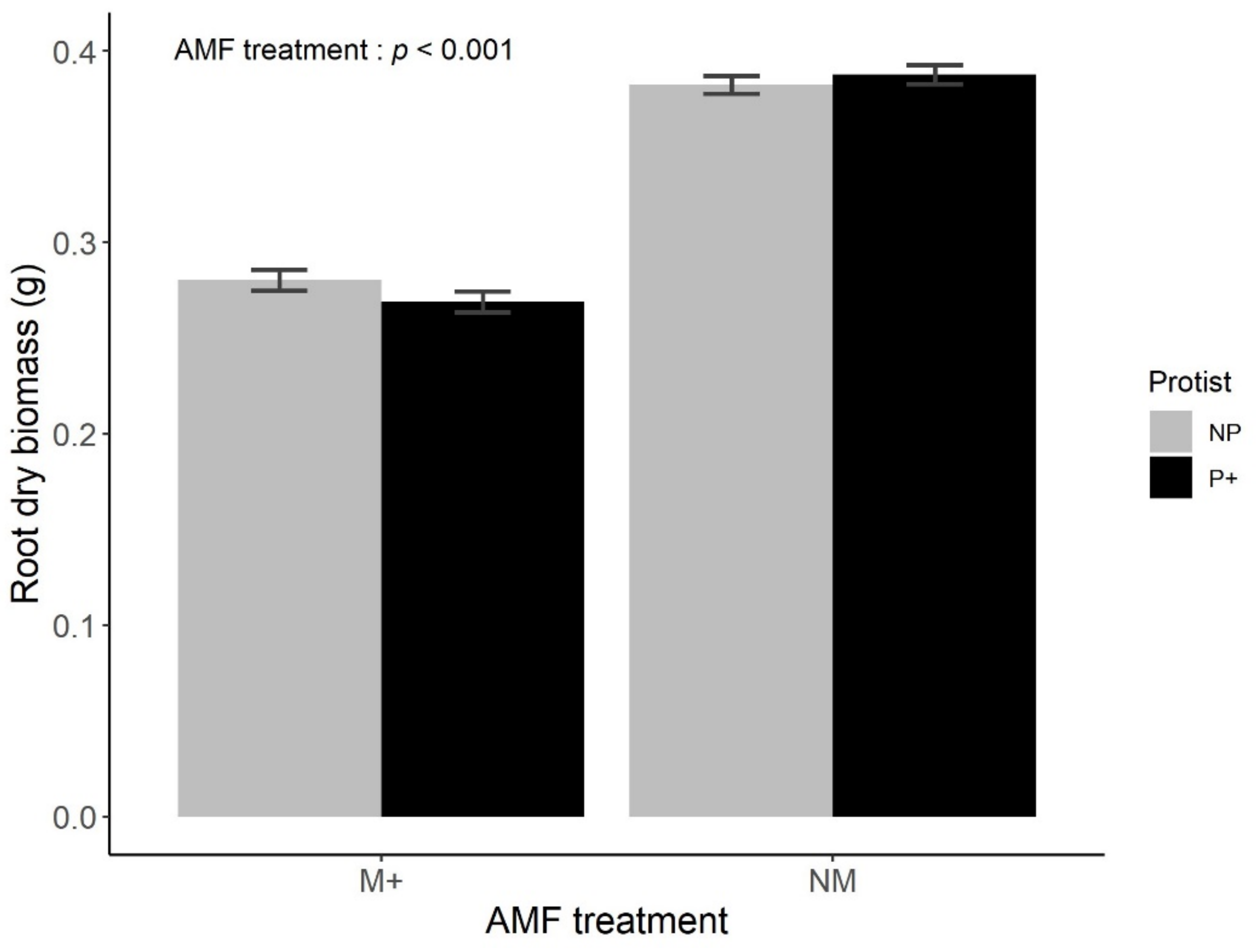

Impact of arbuscular mycorrhizal fungi (AMF) and protist presence on chicory dry root biomass per microcosm. M+, mycorrhizal fungus present; NM, mycorrhizal fungus absent; P+, protists present; NP, protists absent. The results are presented as mean values ± SE (n = 21). Only the effect of AMF treatment (*p*-value shown above the graph) was significant in a two-way ANOVA (see Supporting Information Table S1 for more details).

Supporting Information Figure S6:

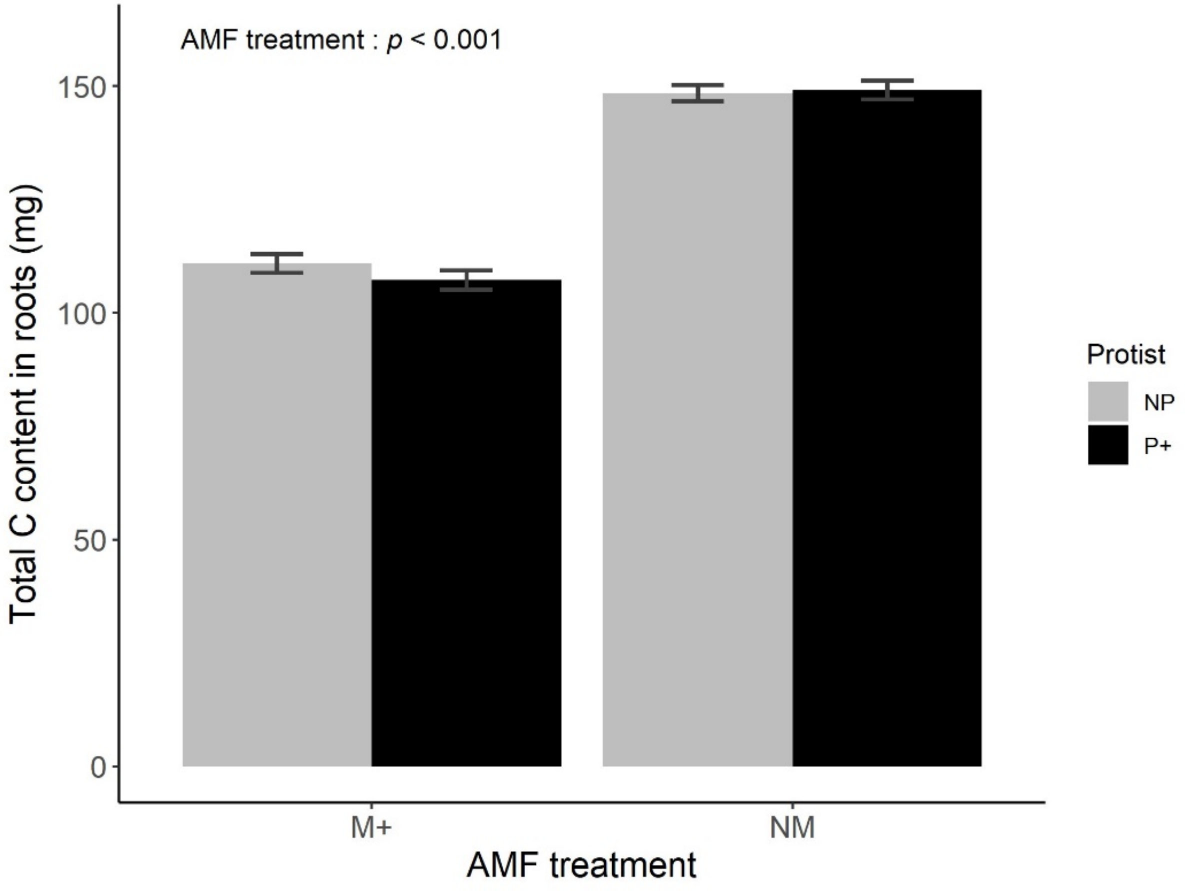

Impact of arbuscular mycorrhizal fungi (AMF) and protist presence on chicory root carbon (C) content per microcosm. M+, mycorrhizal fungus present; NM, mycorrhizal fungus absent; P+, protists present; NP, protists absent. The results are presented as mean values ± SE (n = 21). Only the effect of AMF treatment (*p*-value shown above the graph) was significant in a two-way ANOVA (see Supporting Information Table S1 for more details).

Supporting Information Figure S7:

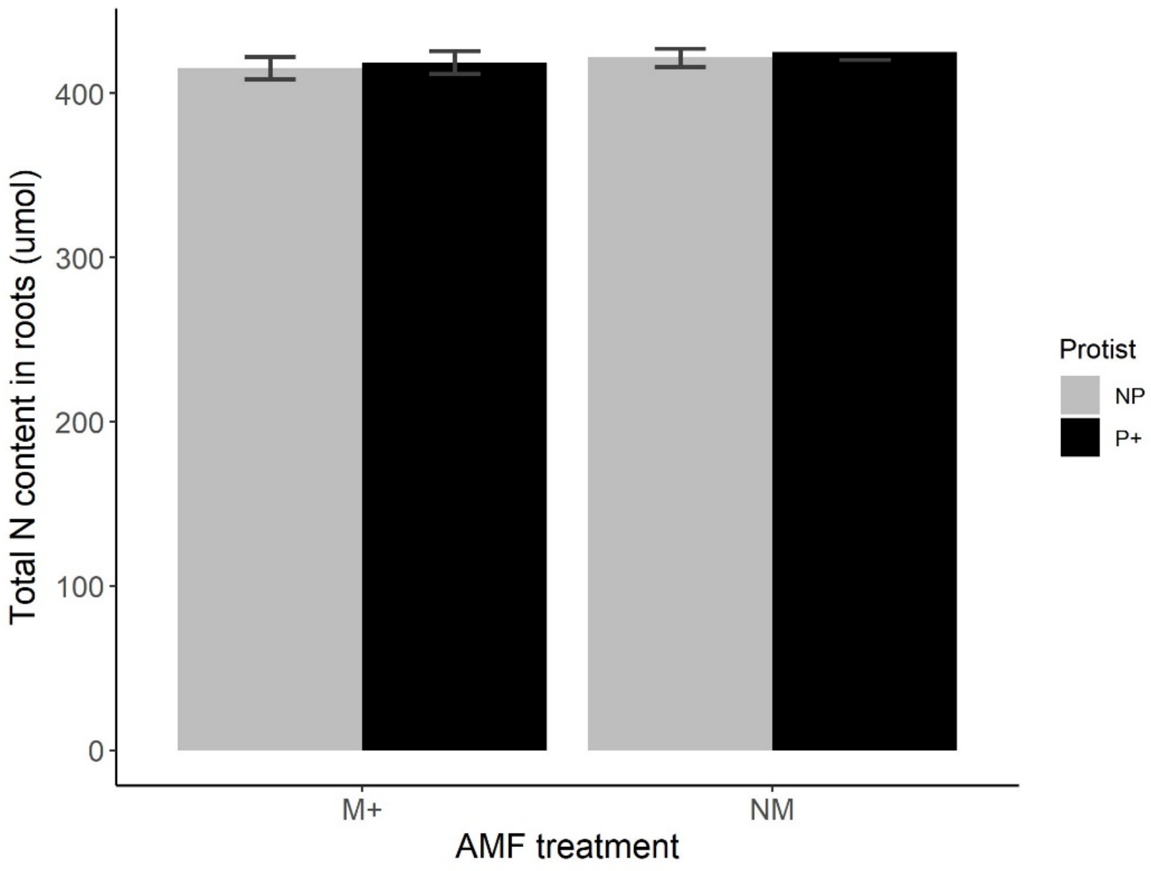

Impact of arbuscular mycorrhizal fungi (AMF) and protist presence on chicory root nitrogen (N) content per microcosm. M+, mycorrhizal fungus present; NM, mycorrhizal fungus absent; P+, protists present; NP, protists absent. The results are presented as mean values ± SE (n = 21). No significant difference due to either of the experimental factors was found in a two-way ANOVA (see Supporting Information Table S1 for more details).

Supporting Information Table S1: Results of ANOVAs for multiple variables tested in this study.

The following factors were included: AMF: arbuscular mycorrhizal fungal presence with two levels – presence or absence; Protist: protist presence with 2 levels – presence or absence; N source: identity of the nitrogen (N) source added to a hyphosphere compartment with 3 levels – ammonium, chitin, or protein. Columns 2–4 refer to root variables and thus two-way ANOVA was used to evaluate the data. For columns 5–7, a linear mixed-effect model with microcosm identity as a random effect was employed to acknowledge a potential co-dependency of analyses of the three root-free compartments from the same microcosm, as detailed in the main text.

	**Variables**					
**Source of variation (factors)**	**Root dry biomass**	**Root total C content**	**Root total N content**	**Bacterial abundance**	**AMF abundance**	**Protist abundance**
AMF	***	***	ns	***	NA	***
Protist	ns	ns	ns	***	*	NA
N sources	NA	NA	NA	***	***	***
AMF × Protist	ns	ns	ns	***	NA	NA
AMF × N sources	NA	NA	NA	***	NA	***
Protist × N sources	NA	NA	NA	***	ns	NA
AMF × Protist × N sources	NA	NA	NA	***	NA	NA

NA, factor is not applicable for the respective analysis (not included in the design of the analysis).

ns, $p \geq 0.05$; *, $0.01 \leq p < 0.05$; ***, $p < 0.001$.

Supporting Information references

Hoysted GA, Field KJ, Sinanaj B, Bell CA, Bidartondo MI, Pressel S. 2023. Direct nitrogen, phosphorus and carbon exchanges between Mucoromycotina 'fine root endophyte' fungi and a flowering plant in novel monoxenic cultures. *New Phytologist* **238**: 70-79.

Read DJ, Haggar J, Magkourilou E, Durant E, Johnson D, Leake JR, Field KJ. 2024. Photosynthate transfer from an autotrophic orchid to conspecific heterotrophic protocorms through a common mycorrhizal network. *New Phytologist* **243**: 398-406.
